# Prognostic and Clinicopathological Significance of the Loss of Expression of Retinoblastoma Protein (pRb) in Oral Squamous Cell Carcinoma: A Systematic Review and Meta-Analysis

**DOI:** 10.3390/cancers15123132

**Published:** 2023-06-09

**Authors:** María López-Ansio, Pablo Ramos-García, Miguel Ángel González-Moles

**Affiliations:** 1 School of Dentistry, University of Granada, 18071 Granada, Spain; lopansmar@correo.ugr.es; 2 Instituto de Investigación Biosanitaria ibs.GRANADA, 18012 Granada, Spain

**Keywords:** retinoblastoma, pRb, oral cancer, prognosis, systematic review, meta-analysis

## Abstract

**Simple Summary:**

Oral cancer has a global incidence of 377,713 new cases and 177,757 deaths annually, according to GLOBOCAN, IARC, and the WHO. Oral squamous cell carcinoma (OSCC) represents around 90% of all oral malignancies and carries a 5-year mortality rate close to 50%. Hanahan and Weinberg introduced a set of defining characteristics that all malignant neoplastic cells are believed to possess, which have had a profound impact on the scientific community. Among these hallmarks of cancer that were identified, the ability to evade growth suppressor signals is of notable relevance in oncogenesis. These actions are essentially carried out through the functions of tumor suppressor genes, the gene RB being singularly relevant, encoding the tumor suppressor retinoblastoma protein (pRb). Loss of pRb expression has a remarkable influence on tumor development, both in its initiation and in its early and late progression; nevertheless, it is striking to note that in oral carcinogenesis there are no meta-analytical studies designed in order to investigate the evidence base on this research topic. This is the first systematic review and meta-analysis, based on 20 studies and 2451 patients with OSCC, demonstrating that the loss of pRb function is a factor associated with improved survival in patients with oral cancer.

**Abstract:**

This systematic review and meta-analysis aims to evaluate the scientific evidence on the implications of retinoblastoma protein (pRb) alterations in oral cancer, in order to determine its prognostic and clinicopathological significance. PubMed, Embase, Web of Science, and Scopus were searched for studies published before February 2022, with no restrictions by publication date or language. The quality of the studies using the Quality in Prognosis Studies tool (QUIPS tool). Meta-analysis was conducted to achieve the proposed objectives, as well as heterogeneity, subgroup, meta-regression, and small study-effects analyses. Twenty studies that met the inclusion criteria (2451 patients) were systematically reviewed and meta-analyzed. Our results were significant for the association between the loss of pRb expression and a better overall survival (HR = 0.79, 95%CI = 0.64–0.98, *p* = 0.03), whereas no significant results were found for disease-free survival or clinico-pathological parameters (T/N status, clinical stage, histological grade). In conclusion, our evidence-based results demonstrate that loss of pRb function is a factor associated with improved survival in patients with OSCC. Research lines that should be developed in the future are highlighted.

## 1. Introduction

Hanahan and Weinberg [1] in 2000 proposed to the scientific community a series of distinctive characteristics that all neoplastic cells should possess, independently of the origin of the tumor tissue. This proposal was completed in 2011 with new characteristics that collectively have received the denomination of hallmarks of cancer [2]. The final proposal of these authors includes a number of hallmarks (sustaining proliferative signaling, evading growth suppressors, resistance to cell death, enabling replicative immortality, angiogenesis, activating invasion and metastasis), two enabling features (genome instability and mutation, and tumor-promoting inflammation), and two emerging hallmarks (deregulating cellular energetics and avoiding immune destruction) [1,2]. The papers by Hanahan and Weinberg [1,2] have reached an enormous impact on the scientific community and have indisputably established lines of research in different types of cancers that have intended to evaluate to what extent these hallmarks are of special value in terms of diagnosis, prognosis or treatment, although it should be noted that to date there is very little evidence on the degree of development of these hallmarks in oral squamous cell carcinoma [3].

Hallmarks of cancer include the ability to evade growth suppressor signals and to resist cell death, which are of notable relevance in oncogenesis [1,2]. These actions are essentially carried out through the functions of tumor suppressor genes, the most important of which are the *RB* gene, encoding the tumor suppressor protein pRb, and the *TP53* gene, encoding p53.

The pRb protein is frequently mutated in human cancers. The physiological functions of pRb include the control of proliferation—by arresting the cell cycle in G1—and promoting cell differentiation and chromosomal stability [4,5], which it achieves essentially, but not exclusively, by sequestration of E2F transcription factors, thus holding them off from their target genes. The loss of *RB* tumor suppressor function has a marked influence on tumor development, both in its initiation and in its early and late progression. The most representative evidence for the importance of loss of pRb function in tumor initiation comes from the genetic study of members of families in which an alteration in *RB* gene alleles is inherited that predisposes to the development of familial retinoblastoma [6,7,8]. It has also been shown in cervical and oropharyngeal cancer, closely associated with HPV infection, that these viruses inactivate pRb through its E7 oncoprotein, this being the mechanism of oncogenic initiation [9,10], and similar findings have been documented for virus-induced hepatocarcinoma [11]. These tumor-initiating actions linked to *RB* loss occur both in stem cells—in which normofunctioning *RB* keeps them in a quiescent state, their usual situation—and in postmitotic differentiated cells—in which *RB* mutation allows them to reintegrate into the cell cycle—and, especially in proliferative progenitor cells (called transitory amplifying cells in the oral epithelium), which constitute an intermediate step between stem cells and postmitotic differentiated cells. It seems likely that transient amplifying cells are the essential source of malignant and premalignant clones in the oral epithelium [12], where loss of pRb could maintain proliferation by preventing their cell cycle exit in G1, which occurs physiologically in these cells after the development of several proliferative cycles [9,10,11,12,13].

Furthermore, some actions of pRb may contribute to progression towards late stages of oncogenesis. This is supported by the fact that most tumor tissues only show *RB* alterations in advanced stages of the disease [14], probably indicating that the conserved function of pRb in neoplastic cells may contribute to the development of paradoxical prosurvival actions. This unexpected role has been supported by the demonstration that loss of pRb promotes cell death [15,16], and through this mechanism the conservation of an intact *RB* gene in tumor cells could prevent apoptosis in order to promote tumor cell survival. Another paradoxical function of pRb could be related to the capability to stimulate autophagy in neoplastic cells, a well-known mechanism that favors cell survival in a hypoxic environment [17,18]. Finally, tumor progression is related to the ability of an altered *RB* gene to induce an undifferentiated status in mutant cells and genomic instability [19].

In spite of the above mentioned factors, it is striking to note that, to date, there are no studies with evidence-based designs, in the form of systematic reviews and meta-analyses, analyzing the role of pRb alterations in oral carcinogenesis. Based on this background, we proposed to perform a systematic review and meta-analysis on the prognostic and clinicopathological implications of pRb alterations in oral cancer in order to determine their prognostic importance and their role as a promoter of the development and progression of this type of tumor.

## 2. Materials and Methods

The systematic review and meta-analysis adhered closely to the criteria established by the Cochrane Prognosis Methods Group [20] and the Cochrane Handbook for Systematic Reviews of Interventions [21]. Reporting of the study was in compliance with PRISMA [22] and MOOSE [23] guidelines.

### 2.1. Protocol

To enhance the transparency, precision, and integrity of our systematic review and meta-analysis, we developed and registered a study protocol with PROSPERO International prospective register of systematic reviews (www.crd.york.ac.uk/PROSPERO), which was assigned the code CRD42022336448, accessed on 10 June 2022. Furthermore, the protocol was reported to minimize bias in accordance with the guidelines of PRISMA-P [24].

### 2.2. Search Strategy

We conducted a comprehensive search for studies published before February 2022 (upper limit) with no lower date limit. The search was performed on MEDLINE (via PubMed), Embase, Scopus, and Web of Science databases, using a combination of thesaurus terms (MeSH and emtree) and free terms to maximize sensitivity. The search strategy is detailed in Table S1 (Supplementary Information). We also manually screened the reference lists of included studies. All references were managed using Mendeley v.1.19.8 (Elsevier, Amsterdam, The Netherlands), which was also used to remove duplicates.

### 2.3. Eligibility Criteria

Our inclusion criteria for this study included original primary-level studies that evaluated pRb expression in samples from OSCC without any restrictions on language, publication date, follow-up periods, geographical area, age, or sex. The analysis of the association of the loss of pRb expression with at least one of the following prognostic and/or clinicopathological variables was also required: overall survival (OS), disease-free survival (DFS), tumor size, N status, clinical stage, or histological grade. OS was defined as the time elapsed from the date of diagnosis/surgery to the date of death by any cause. DFS was defined as the time elapsed from diagnosis/surgery to the detection of locoregional or distant recurrence or to death without recurrence. We included any study that used the terms OS/DFS or other terms complying with our precedent definitions since there is a lack of international consensus standards to define survival endpoints in oncology research.

Exclusion criteria for our study included retracted articles, preclinical research (in vitro research or in vivo animal experimentation), case reports, editorials, letters, meeting abstracts, personal opinions, comments, or book chapters, or secondary/tertiary-evidence level studies (systematic reviews, meta-analyses, scoping reviews, umbrella, or overviews of reviews, etc.). We also excluded squamous cell carcinomas from anatomic areas distinct to the oral cavity, and/or tumors of different histopathological lineage, evaluation of pRb genomic alterations (e.g., mutations, gene amplification or deletion, polymorphisms, etc.), those with no analysis of the main prognostic or clinicopathological outcomes of interest, lack of or insufficient data for the estimation of statistical effect size measures with their corresponding confidence intervals, and inter-study overlapping populations. Potential inter-study overlapping populations were determined by verifying the authors’ names, affiliations, source of patients, and recruitment periods. Under these circumstances, only the reports reporting more complete datasets were finally included.

### 2.4. Study Selection Process

Two blinded authors (MLA and PRG) independently applied eligibility criteria, discrepancies were resolved by consensus with a third author (MAGM). The article selection process was conducted in two phases: phase-I involved screening titles and abstracts, while phase-II involved reading the complete records. The evaluators underwent training and calibration and performed a joint initial screening round of 50 papers each. An optimal inter-agreement score of 99.89% was achieved. Inter-rater reliability was also measured using Cohen’s kappa statistic, which showed almost perfect agreement (κ = 0.94).

### 2.5. Data Extraction

After completing full-text reading, both authors (MLA and PRG) independently extracted data from the selected articles in a standardized manner using Excel software (v.16/2018, Microsoft, Redmond, WA, USA). The extracted datasets were cross-checked jointly, and any discrepancies were resolved by consensus. The data collected included information on the first author, language and publication date, country, sample size, anatomical subsite of cancer, sex and age of patients, tobacco, areca nut and alcohol consumption, recruitment and follow-up period, study design, immunohistochemical methods (i.e., anti-pRb antibody, dilution, incubation time, and temperature), cut-off point for positivity, scoring system, subcellular pRb location pattern, and relative frequency of the loss of pRb expression. Additionally, data required for analyzing the outcomes were also recorded for survival (OS and DFS) and clinicopathological variables (T-status [T3/T4 vs. T1/T2], N-status [N+ vs. N−], clinical stage [III/IV vs. I/II], and histological grade [II/III vs. I]).

### 2.6. Evaluation of Quality and Risk of Bias

Two authors (MLA and PRG) critically appraised the methodological quality and risk of bias (RoB) across primary-level studies using the Quality in Prognosis Studies (QUIPS) tool (developed by members of the Cochrane Prognosis Methods Group [25]). The following six potential bias domains were explored: (1) study participation; (2) study attrition; (3) prognostic factor measurement; (4) outcome measurement; (5) study confounding; (6) statistical analysis/reporting. The RoB was considered low, moderate, or high for each domain. Finally, an overall score was also estimated based on a method previously described by our research group [26,27,28,29], in order to statistically analyze the influence of the methodological quality of primary-level studies on our meta-analytical results, thus estimating pooled effect sizes adjusted for risk of bias.

### 2.7. Statistical Analysis

We analyzed the loss of pRb expression as a dichotomous categorical variable, using cut-off values from primary-level studies. Odds ratios (OR) with their corresponding 95% confidence intervals (CI) were estimated as effect size metrics for the meta-analysis of clinicopathological variables. Hazard ratios (HR) and 95%CI were managed for the meta-analysis of prognostic variables due to their time-to-event nature [30]. When authors reported effect size metrics in their survival analyses, these were directly extracted from the primary-level studies. If HR and/or 95%CI were not explicitly provided by the authors, we calculated them using the methods described by Parmar et al. [31] and Tierney and colleagues [30]. When a study only reported Kaplan–Meier curves, we extracted the data from the curves using Engauge Digitizer 4.1 software (open-source digitizing software developed by M. Mitchell). All meta-analyses were conducted using the inverse-variance method under a random-effects model (based on the DerSimonian and Laird method), considering a *p*-value < 0.05 as statistically significant. This approach was a priori planned in our study protocol, in order to account for the possibility that are different underlying effects among study subpopulations (mainly due to, hypothetically, the differences among experimental immunohistochemical methods and differences in geographical areas). Forest plots were constructed in all meta-analyses performed, in order to graphically represent the effect sizes and for subsequent visual inspection analysis.

Heterogeneity between studies was assessed using the χ^2^-based Cochran’s Q test. Given the low statistical power of the Q-test, *p* < 0.10 was considered significant. We also applied Higgins I^2^ statistic to estimate what proportion of the variance in observed effects reflects variation in true effects, rather than sampling error. The percentage of inter-study heterogeneity was quantified considering values of 50–75% as moderate-to-high degree of inconsistency [32,33]. Preplanned subgroup meta-analyses (by geographical area, immunohistochemical methods and risk of bias) were performed to identify potential sources of heterogeneity. Furthermore, additional univariable random-effect meta-regression analyses were conducted, using the restricted maximum likelihood (REML) method, to explore the potential effect of additional study covariates (i.e., follow up period, age, sex, and clinical stage) [34]. Considering the low number of studies with data available for meta-regression analyses, the *p*-values were re-calculated using a permutation test based on Monte Carlo simulations [35]. To obtain sufficient precision, the number of permutations was 10,000 [36]. Weighted bubble plots were also constructed to graphically represent the fitted meta-regression lines.

Finally, small-study effects analyses were carried out in order to identify potential biases, such as publication bias, and to test the reliability and robustness of our meta-analytical results. Funnel plots were constructed and the Egger regression test was run (performing a linear regression of the effect estimates on their standard errors, weighting by 1/[variance of the effect estimate]), considering a *p*_Egger_-value < 0.10 as significant [37]. Stata software was used for all statistical analyses (v.16.1, Stata Corp, College Station, TX, USA).

## 3. Results

### 3.1. Results of the Literature Search

**Figure 1:**
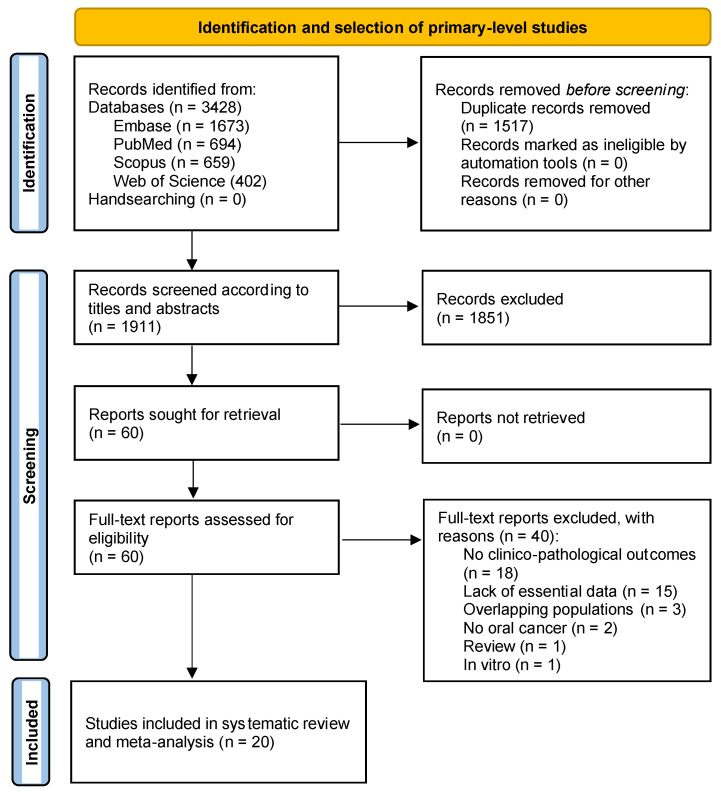
Flow diagram showing the identification and selection process of target studies, analyzing the prognostic and clinicopathological significance of the loss of expression of retinoblastoma protein (pRb) in patients suffering from OSCC.

The flow diagram in Figure 1 depicts the process of identification, screening, and selection of primary-level studies. In total, 3428 records were retrieved: 1673 from Embase, 694 from PubMed, 659 from Scopus, and 402 from Web of Science.

After the removal of duplicates, 1911 records were screened according to titles and abstracts, leaving a sample of 60 papers for full text evaluation (the studies excluded and their exclusion criteria were listed in the List S1, Supplementary Information). Finally, 20 studies meeting all eligibility criteria were included for qualitative evaluation and meta-analysis [38,39,40,41,42,43,44,45,46,47,48,49,50,51,52,53,54,55,56,57].

### 3.2. Study Characteristics

Table 1 summarizes the main characteristics of our study sample, and Table S2 (Supplementary Information) exhibits in detail the main variables gathered. These 20 primary-level studies recruited a total of 2451 patients, ranging between 9 and 784. All studies harbored a homogeneous study design, observational retrospective cohorts, applying immunohistochemistry and assessing the prognostic value of the loss of pRb expression consistently in the cell nucleus. In relation to the experimental methods and laboratory conditions, the Clone IF8 was the anti-pRb antibody most frequently used (n = 4). Most studies processed their antibodies at dilutions from 1:5 to 1:50 (n = 6), at overnight incubation (n = 8).

Table 1 represents a summary of the main characteristics of the study. Table S2 (Supplementary Information) exhibits in detail the characteristics of each primary-level study included in this systematic review and meta-analysis.

### 3.3. Qualitative Evaluation

**Figure 2:**
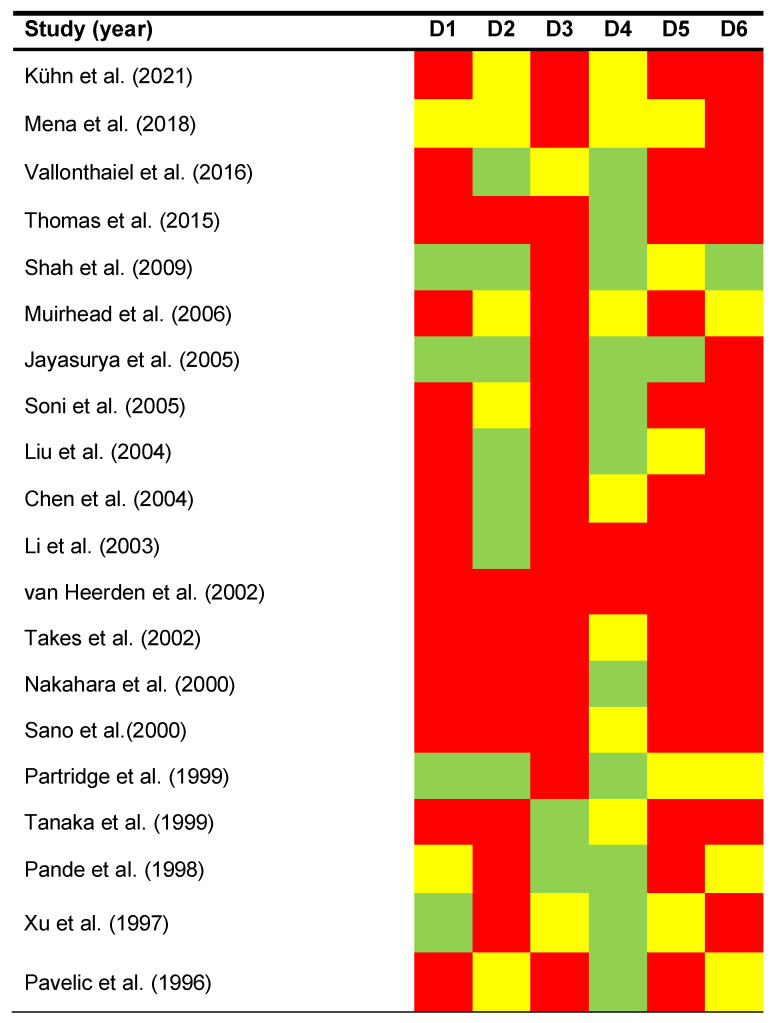
Quality plot depicting the potential RoB across primary-level studies, assessed using the Quality in Prognosis Studies tool (QUIPS) developed by Cochrane Prognosis Methods Group. RoB was critically judged as low (green), moderate (yellow), or high (red) for each domain.

The qualitative analysis was carried out using the QUIPS tool, which evaluates potential sources of bias in six domains (Figure 2):

*Study participation*. RoB was judged as high in 70% of the studies reviewed, moderate in 10% and low in 20%. Studies that provided an inadequate description of their samples (sex and age of patients, oral cancer subsites, etc.) or clinical setting (site and recruitment period) were considered potentially biased.

*Study attrition*. RoB was high in 35% of the studies, moderate in 30%, and low in 35%. Some studies did not report essential information on the follow-up period (i.e., mean ± SD, median, IQR, and/or range), whereas most studies did not report data on drop-out rates.

*Prognostic factor measurement*. RoB was high in 80% of the studies, moderate in 10%, and low in 10%. The most relevant potential bias was the lack of reporting of the anti-pRb antibodies used. Inadequate design of cut-off points and unclear scoring systems for pRb expression were also judged as serious sources of potential bias.

*Outcome measurement*. RoB was high in 10% of the studies, moderate in 35% and low in 55%. The most frequent potential biases were the failure to define survival parameters—something which is imperative due to the lack of international consensus on survival endpoints in cancer research—and the lack of reporting of the staging system used.

*Study confounding*. RoB was high in 70% of the studies, moderate in 25%, and low in 5%. The most frequent potential bias was the failure to account for confounding factors in the study design.

*Statistical analysis and reporting*. RoB was high in 75% of the studies, moderate in 20%, and low in 5%. The most serious potential biases were inadequate statistical analyses and manifest reporting errors, adding to misleading results and conclusions. Frequently, studies did not compute effect sizes which are necessary to estimate the impact of the study variables (e.g., HR with 95%CI).

### 3.4. Quantitative Evaluation (Meta-Analysis)

#### 3.4.1. Association between the Loss of pRb Expression and Prognostic Variables

**Figure 3:**
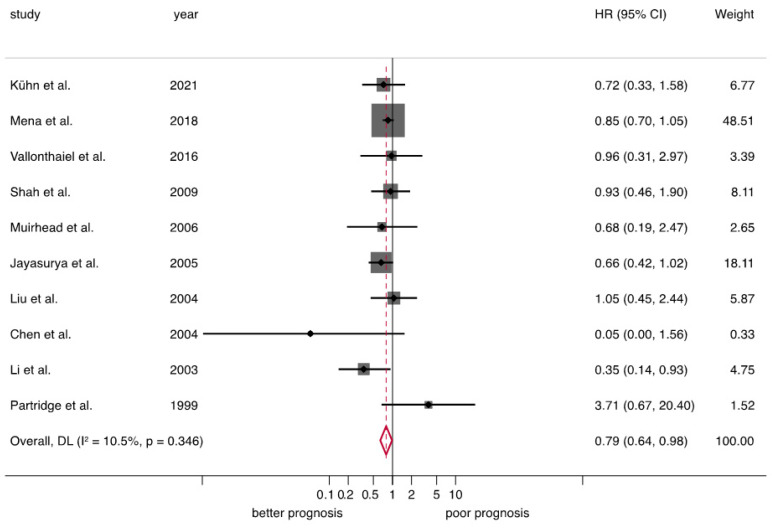
Forest plot graphically representing the meta-analysis on the association between the loss of expression of retinoblastoma protein (pRb) and overall survival in patients suffering from OSCC. Random-effects model, inverse-variance weighting (based on the DerSimonian and Laird method). A HR > 1 suggests that the loss of pRb is associated with poor prognosis. Diamonds indicate the pooled HRs with their corresponding 95%CIs. Abbreviations: pRb, retinoblastoma protein; OS, overall survival; OSCC, oral squamous cell carcinoma; HR, hazard ratio; CI, confidence intervals.

*Overall survival (OS).* Significant results were found for the loss of pRb expression and better OS (HR = 0.79, 95%CI = 0.64–0.98, *p* = 0.03), obtaining homogeneous results across primary-level studies (heterogeneity: *p* = 0.35, I^2^ = 10.5%) (Table 2 and Figure 3).

*Disease-free survival (DFS).* Significant results were not found for the loss of pRb expression and DFS (HR = 1.09, 95%CI = 0.59–2.10, *p* = 0.79), and a considerable degree of heterogeneity was observed (*p* = 0.02, I^2^ = 67.5%) (Table 2 and Figure S11, Supplementary Information).

#### 3.4.2. Association between the Loss of pRb Expression and Clinico-Pathological Variables

The loss of pRb expression was not significantly associated with the clinico-pathological variables investigated (T status: OR = 1.89, 95CI% = 0.97–3.69, *p* = 0.06; N status: OR = 1.25, 95%CI = 0.76–2.10, *p* = 0.40; clinical stage: OR = 1.25, 95%CI = 0.65–2.39, *p* = 0.50; histological grade: OR = 0.95, IC 95% = 0.67–1.34, *p* = 0.03; Table 2 and Figures S12–S15, Supplementary Information).

### 3.5. Quantitative Evaluation (Secondary Analyses)

*Meta-analysis of subgroups.* Effect sizes did not significantly vary across the subgroups investigated (i.e., stratified by geographical area, anti-pRb antibody, antibody dilution, incubation time and temperature, cut-off point, and overall risk of bias across primary-level studies), all of them showing a relatively stable and similar prognostic behavior (Table 2 and Figures S1–S7, Supplementary Information).

*Meta-regression analysis.* The potential effect of the study covariates sex, age, and follow up on the association between the loss of pRb expression and OS was analyzed. No significant differences were found, potentially ruling out these covariates as sources of heterogeneity (Table 2 and Figures S8–S10, Supplementary Information). The rest of covariates (i.e., tobacco, alcohol, and betel quid) were not included in meta-regressions due to the low number of observations reported in primary-level studies.

*Small-study effects analysis.* Visual inspection analysis of the asymmetry of the funnel plots constructed and the statistical tests conducted for the same purpose confirmed the absence of small-study effects for all variables (OS: *p*_Egger_ = 0.617; DFS: *p*_Egger_ = 0.224; T status: *p*_Egger_ = 0.290; N status: *p*_Egger_= 0.584; clinical stage: *p*_Egger_= 0.260), except for histological grade (*p*_Egger_= 0.068) for which biases, e.g., publication bias, could not be ruled out (Figure S16–S21, Supplementary Information).

## 4. Discussion

The main results of our meta-analysis on 20 primary-level studies and 2451 patients with OSCC point out that pRb expression is a factor which improves survival of patients with oral cancer (HR = 0.79, 95CI% = 0.64–0.98, *p* = 0.03), this data indicates that patients with oral cancer who lose pRb expression survive 1.27 times more than patients who maintain gene and protein function. This result could be initially considered paradoxical, since it reflects that loss of function of a tumor suppressor gene improves survival. We propose that a hypothetical explanation for this observation could be related to the effect that HPVs exert on the improved survival of oral cancer patients, knowing that an essential pathway of HPV-linked oncogenesis is established through the suppression of pRb actions by its E7 oncoprotein in high-risk HPVs (16 and 18). Thus, perhaps it is not pRb downregulation which leads to improved oral cancer survival, but the fact that this downregulation is associated with the oncogenic actions of HPVs, which inactivate pRb and are well-known to generate oral carcinomas with a better prognosis [58,59].

This hypothesis could be confirmed with evidence base if studies were available to compare the frequency of pRb downregulation in HPV(+) OSCC vs. HPV(−) OSCC, analyzing their prognostic differences including survival. Our hypothesis would be supported by the finding of a significant pRb downregulation in HPV(+) OSCC, which would also show a better survival than the rest. Confirmation of this hypothesis would finally be obtained if HPV(−) OSCCs did not differ in patient survival according to their pRb status (positive-pRb vs. loss-pRb). This would indicate that the improvement in survival is essentially influenced by HPV infection. However, the data published on this subject in primary level studies are scarce and not conclusive. Only 9 primary level studies have analyzed pRb status and its relation to HPV infection in OSCC and none of them provide survival data [44,45,60,61,62,63,64,65,66]. Loss of pRb in HPV(+) OSCC has been studied in only three series with discordant results (0%, 50%, and 66.67%) [44,62,66]. On the contrary the presence of HPV infection in OSCC patients who have lost pRb function (pRb-negatively) has only been addressed in two studies with results of 12% and 33.33% [45,60], respectively. Furthermore, the magnitude of association demonstrates in 2 out of 6 studies that the risk of inactivating pRb is significantly higher in HPV(+) OSCCs [45,60,61,63,64,65]. As can be seen, although there are suggestive data in this regard, the scientific evidence is very limited, among other reasons because of the lack of survival analysis and thus, for the moment, the best prognosis of oral carcinomas that lose pRb expression is of unknown cause.

A second relevant result of our systematic review and meta-analysis refers to the fact that pRb downregulation was not associated with any of the classical clinicopathological parameters with prognostic implications in oral cancer (T status, N status, clinical stage, etc.), which could indicate that alterations in this tumor suppressor essentially influence the phases of tumor initiation.

Based on our qualitative assessment, performed by applying QUIPS tool [25] (developed by Cochrane Prognostic Methods Group [20]), although the studies in our meta-analysis had similar experimental designs, not all were conducted with the same methodological rigor. The domains third (*prognostic factor measurement)* and fifth (*study confounding)* harbored a higher risk of potential bias than the rest. Therefore, future studies should improve their methodological quality by increasing the transparency in the reporting of the experimental methods carried out to investigate the loss of pRb expression, as well as improving the design of scoring systems and application of cut-off points; and be more rigorous in the design and control of potentially confounding factors, which are essential in studies of an observational nature. These methodological recommendations derived from the present systematic review should be followed in order to improve and standardize future research.

As potential limitations of our meta-analysis, the number of observations was low or insufficient for several variables with potential prognostic implications that could not be quantitatively evaluated (e.g., tobacco, alcohol, and betel quid consumption). This is actually an inherent limitation of the primary-level studies included in our systematic review and meta-analysis, and as previously reflected, future studies should report and publish their datasets in a more rigorous way. Strengths of our meta-analysis include robust, statistically homogeneous results, which are not affected by publication bias, and which could be ruled out. In addition, this is the first meta-analysis researching the prognostic implications of pRb in oral cancer, which presents additional value for the literature, as well as the identification of evidence gaps and methodological recommendations for future research.

## 5. Conclusions

In conclusion, our evidence-based results demonstrate that loss of pRb functions is a factor associated with improved survival in oral squamous cell carcinoma patients. This observation could be attributed to the oncogenic effects of HPV that are exerted by inactivation of pRb. These viruses have been shown to generate better prognostic oral squamous cell carcinoma. Future research should confirm this hypothesis as, to date, there are limited primary-level studies on this topic.

## Figures and Tables

**Table 1:** Summarized characteristics of the study sample.

Total	20 Studies
Year of publication	1996–2021
Total patients (range)	2451 (9–784)
Study design	
Retrospective cohort	20 studies
Experimental methods for pRb expression determination	
Immunohistochemistry	20 studies
Anti-pRb antibody	
Clone IF8	4 studies
Clone G3-245	2 studies
Other	5 studies
Not reported	9 studies
Anti-pRb antibody dilution	
1:5–1:50	6 studies
1:100	5 studies
1:150–1:700	4 studies
Not reported	5 studies
Anti-pRb antibody incubation time	
Overnight	8 studies
1 h or less	2 studies
Not reported	10 studies
Anti-pRb antibody incubation temperature	
4 °C	7 studies
Room temperature	2 studies
Not reported	11 studies
Geographical region	
Asian countries	11 studies
Non-Asian countries	9 studies

**Table 2:** Meta-analyses of prognostic and clinicopathological significance of the loss of pRb expression in OSCC.

					Pooled Data		Heterogeneity	
Meta-Analyses	No. of Studies	No. of Patients	Stat. Model	Wt	ES (95% CI)	*p*-Value	*P_het_*	*I^2^* (%)
**SURVIVAL PARAMETERS**								
**Overall survival**								
Loss of pRb expression (all) ^a^	10	1636	REM	D–L	HR = 0.79 (0.64–0.98)	0.03	0.35	10.5
Subgroup analysis by geographical area ^b^						0.98 ^c^		
Asian	5	599	REM	D–L	HR = 0.77 (0.55–1.07)	0.12	0.47	0.0
Non-Asian	5	1037	REM	D–L	HR = 0.77 (0.49–1.19)	0.23	0.17	37.6
Subgroup analysis by anti-pRb antibody ^b^						0.68 ^c^		
Clone IF8	1	340	-	-	HR = 0.66 (0.42–1.02)	0.07	-	-
Clone G3-245	1	55	-	-	HR = 1.05 (0.45–2.44)	0.91	-	-
Other	2	122	REM	D–L	HR = 1.36 (0.28–6.52)	0.70	0.09	65.8
Not reported	6	1119	REM	D–L	HR = 0.78 (0.58–1.05)	0.10	0.33	12.7
Subgroup analysis by anti-pRb antibody dilution ^b^						0.76 ^c^		
1:0–50	3	224	REM	D–L	HR = 1.07 (0.51–2.25)	0.85	0.27	24.4
1:100	1	60	-	-	HR = 0.96 (0.31–2.97)	0.94	-	-
1:150–700	3	142	REM	D–L	HR = 0.78 (0.38–1.59)	0.49	0.27	24.6
Not reported	3	1210	REM	D–L	HR = 0.70 (0.49–1.01)	0.06	0.14	49.6
Subgroup analysis by anti-pRb antibody incubation time ^b^						0.97 ^c^		
1 h or less	2	123	REM	D–L	HR = 0.71 (0.36–1.38)	0.31	0.94	0.0
Overnight	3	535	REM	D–L	HR = 0.75 (0.52–1.07)	0.11	0.65	0.0
Not reported	5	978	REM	D–L	HR = 0.79 (0.43–1.47)	0.47	0.07	54.2
Subgroup analysis by anti-pRb antibody incubation temperature ^b^						0.97 ^c^		
4 °C	3	535	REM	D–L	HR = 0.75 (0.52–1.07)	0.11	0.65	0.0
Room temperature	2	123	REM	D–L	HR = 0.71 (0.36–1.38)	0.31	0.94	0.0
Not reported	5	978	REM	D–L	HR = 0.79 (0.43–1.47)	0.47	0.07	54.2
Subgroup analysis by cut-off point ^b^						0.74 ^c^		
<10	2	123	REM	D–L	HR = 0.71 (0.36–1.38)	0.31	0.94	0.0
10	2	190	REM	D–L	HR = 0.98 (0.57–1.68)	0.94	0.83	0.0
>10	5	983	REM	D–L	HR = 0.77 (0.39–1.50)	0.44	0.08	53.0
Intensity-based	1	340	-	-	HR = 0.66 (0.42–1.03)	0.07	-	-
Subgroup analysis by overall risk of bias in primary-level studies ^b^						0.14 ^c^		
Low RoB	1	340	-	-	HR = 0.66 (0.42–1.03)	0.07	-	-
Moderate RoB	5	1078	REM	D–L	HR = 0.88 (0.73–1.06)	0.19	0.55	0.0
High RoB	4	218	REM	D–L	HR = 0.53 (0.31–0.92)	0.03	0.38	1.8
Univariable meta-regressions by study design and patients’ characteristics ^d^								
Follow up (months, mean)	10	1636	random-effects meta-regression		Coef = −0.005 (−0.052 to 0.042)	0.69 ± 0.005 ^e^	het_explained_ = −1320% ^f^	
Sex (proportion of males, %)	7	1417	random-effects meta-regression		Coef = 0.005 (−0.021 to 0.030)	0.94 ± 0.002 ^e^	het_explained_ = 0.00% ^f^	
Age (years, mean)	8	1472	random-effects meta-regression		Coef = −0.010 (−0.058 to 0.039)	0.53 ± 0.005 ^e^	het_explained_= 0.00% ^f^	
Clinical stage (proportion of stage-III/IV patients, %)	4	233	-		-	-	-	
Tobacco consumption (proportion of smokers, %)	4	1303	-		-	-	-	
Areca nut/Betel quid consumption (proportion of chewers, %)	1	55	-		-	-	-	
Alcohol consumption (% of patients with positive habit)	2	828	-		-	-	-	
**Disease-free survival**								
Loss of pRb expression (all) ^a^	5	799	REM	D–L	HR = 1.09 (0.59–2.02)	0.79	0.02	67.5
**CLINICO-PATHOLOGICAL CHARACTERISTICS**								
**T status**								
Loss of pRb expression (all) ^a^	8	756	REM	D–L	OR = 1.89 (0.97–3.69)	0.06	0.003	67.9
**N status**								
Loss of pRb expression (all) ^a^	11	786	REM	D–L	OR = 1.25 (0.76–2.10)	0.40	0.06	43.4
**Clinical Stage**								
Loss of pRb expression (all) ^a^	5	453	REM	D–L	OR = 1.25 (0.65–2.39)	0.50	0.18	36.6
**Histological grade**								
Loss of pRb expression (all) ^a^	11	812	REM	D–L	OR = 0.95 (0.67–1.34)	0.77	0.42	2.8

Abbreviations: Stat., statistical; Wt, method of weighting; ES, effect size estimation; HR, hazard ratio; OR, odds ratio; CI, confidence intervals; REM, random-effects model; D–L, DerSimonian and Laird method; OSCC, oral squamous cell carcinoma; RoB, risk of bias; pRb, retinoblastoma protein. ^a^—Meta-analysis of aggregate (summary) data. ^b^—Subgroup meta-analyses. ^c^—Test for between-subgroup differences. ^d^—Meta-regression analysis of the potential effect of study covariates on the association between the loss of pRb and overall survival in OSCC. A meta-regression coefficient > 0 indicates a greater impact of covariates on poor prognosis. ^e^—*p*-value ± standard error recalculated after 10,000 permutations based on Montecarlo simulations. ^f^—Proportion of between-study variance explained (adjusted R^2^ statistic) using the residual maximum likelihood (REML) method. A negative number for proportion of heterogeneity reflects no heterogeneity.

## Data Availability

The data that supports the findings of this study are available in the Supplementary Materials of this article.

## Supplementary Materials

The following supporting information can be downloaded at: https://www.mdpi.com/article/10.3390/cancers15123132/s1, Table S1. Search strategy for each database, number of results, and execution date. Table S2. Characteristics of analyzed studies (n = 20). Figure S1. Forest plot graphically representing the stratified analysis by geographical area on the association between the loss of pRb expression and overall survival in patients with OSCC. Figure S2. Forest plot graphically representing the stratified analysis by anti-pRb antibody on the association between the loss of pRb expression and overall survival in patients with OSCC. Figure S3. Forest plot graphically representing the stratified analysis by anti-pRb antibody dilution on the association between the loss of pRb expression and overall survival in patients with OSCC. Figure S4. Forest plot graphically representing the stratified analysis by anti-pRb antibody incubation time on the association between the loss of pRb expression and overall survival in patients with OSCC. Figure S5. Forest plot graphically representing the stratified analysis by anti-pRb antibody incubation temperature on the association between the loss of pRb expression and overall survival in patients with OSCC. Figure S6. Forest plot graphically representing the stratified analysis by cut-off point on the association between the loss of pRb expression and overall survival in patients with OSCC. Figure S7. Forest plot graphically representing the stratified analysis by overall RoB in primary-level studies, on the association between the loss of pRb expression and overall survival in patients with OSCC. Figure S8. Bubble plot graphically representing the univariable meta-regression analysis of the potential effect of follow up period (expressed in months, in x-axis) on the association between the loss of pRb expression and overall survival in patients with OSCC (using HR as effect size measure, in y-axis). Figure S9. Bubble plot graphically representing the univariable meta-regression analysis of the potential effect of sex (% of males) on the association between the loss of pRb expression and overall survival in patients with OSCC. Figure S10. Bubble plot graphically representing the univariable meta-regression analysis of the potential effect of age (mean age of patients, expressed in years) on the association between the loss of pRb expression and overall survival in patients with OSCC. Figure S11. Forest plot graphically representing the meta-analysis on the association between the loss of pRb expression and DFS in patients with OSCC. Figure S12. Forest plot graphically representing the meta-analysis on the association between the loss of pRb expression and T status (T3/T4 vs. T1/T2) in patients with OSCC. Figure S13. Forest plot graphically representing the meta-analysis on the association between the loss of pRb expression and N status (positive metastatic lymph nodes vs. negative) in patients with OSCC. Figure S14. Forest plot graphically representing the meta-analysis on the association between the loss of pRb expression and clinical stage (III/IV vs. I/II) in patients with OSCC. Figure S15. Forest plot graphically representing the meta-analysis on the association between the loss of pRb expression and histological grade (poorly-moderate vs. well-differentiated carcinomas) in patients with OSCC. Figure S16. A funnel plot of estimated logHRs against their standard errors, graphically representing the analysis of small-study effects on the association between the loss of pRb expression and overall survival in OSCC. Figure S17. A funnel plot of estimated logHRs against their standard errors, graphically representing the analysis of small-study effects on the association between the loss of pRb expression and DFS in OSCC. Figure S18. A funnel plot of estimated logORs against their standard errors, graphically representing the analysis of small-study effects on the association between the loss of pRb expression and T status in OSCC. Figure S19. A funnel plot of estimated logORs against their standard errors, graphically representing the analysis of small-study effects on the association between the loss of pRb expression and N status in OSCC. Figure S20. A funnel plot of estimated logORs against their standard errors, graphically representing the analysis of small-study effects on the association between the loss of pRb expression and clinical stage in OSCC. Figure S21. A funnel plot of estimated logORs against their standard errors, graphically representing the analysis of small-study effects on the association between the loss of pRb expression and histological grade in OSCC. List S1: List of full-text articles excluded with reasons.
