# Supplementary material for: Prognostic and Clinicopathological Significance of the Loss of Expression of Retinoblastoma Protein (pRb) in Oral Squamous Cell Carcinoma: A Systematic Review and Meta-Analysis
Source: Cancers (Basel). 2023 Jun 9;15(12):3132. doi: 10.3390/cancers15123132 (PMC10296286; doi:10.3390/cancers15123132)
Supplement: Supplementary file 1 [file cancers-15-03132-s001.zip › cancers-2418270-supplementary.pdf]

Supplementary Information to the Manuscript

Prognostic and clinicopathological significance of the loss of expression of retinoblastoma protein (pRb) in oral squamous cell carcinoma: a systematic review and meta-analysis.

Lopez-Ansio M[1,2], Ramos-Garcia P[1,2,*], and Gonzalez-Moles MA[1,2,*]

1- School of Dentistry, University of Granada, Granada, Spain

2- Biohealth Research Institute Ibs.Granada, Granada, Spain

***Corresponding Authors:**

Pablo Ramos-Garcia

Miguel Ángel González Moles

Oral Medicine Department, School of Dentistry, University of Granada,

Granada, Paseo de Cartuja s/n, 18071 Granada, Spain.

Tel.:+34958243804; fax:+34958240908. E-mail: pabloramos@ugr.es; magonzal@ugr.es

Table of contents

1. Search strategy. Table S1. 3
2. Characteristics of analyzed studies. Table S2 4
3. Meta-analysis on the loss of pRb expression and overall survival in OSCC 6
    3.1 Subgroup meta-analysis by geographical area 6
    3.2 Subgroup meta-analysis by anti-pRb antibody 7
    3.3 Subgroup meta-analysis by anti-pRb antibody dilution 8
    3.4 Subgroup meta-analysis by anti-pRb antibody incubation time 9
    3.5 Subgroup meta-analysis by anti-pRb antibody incubation temperature 10
    3.6 Subgroup meta-analysis by cut-off point 11
    3.7 Subgroup meta-analysis by overall risk of bias in primary-level studies 12
    3.8 Univariable meta-regression on the effect of follow up 13
    3.9 Univariable meta-regression on the effect of sex 14
    3.10 Univariable meta-regression on the effect of age 15
4. Meta-analysis on the loss of pRb expression and DFS in OSCC 16
5. Meta-analysis on the loss of pRb expression and T status 17
6. Meta-analysis on the loss of pRb expression and N status 18
7. Meta-analysis on the loss of pRb expression and clinical stage 19
8. Meta-analysis on the loss of pRb expression and histological grade 20
9. Analysis of small-study effects. 21
    9.1 Loss of pRb expression and overall survival in OSCC 21
    9.2 Loss of pRb expression and DFS in OSCC 22
    9.3 Loss of pRb expression and T status in OSCC 23
    9.4 Loss of pRb expression and N status in OSCC 24
    9.5 Loss of pRb expression expression and clinical stage in OSCC 25
    9.6 Loss of pRb expression and histological grade in OSCC 26
10. List of excluded studies with reasons 27

1. Search strategy

Table S1. Search strategy for each database, number of results, and execution date.

Database	Query/Search Strategy	Results/ Items founds	Search time limits
PubMed	("Retinoblastoma"[Mesh] OR "retinoblastoma"[All Fields] OR "rb"[All Fields] OR "prb"[All Fields] OR "osrc"[All Fields] OR "pp110"[All Fields] OR "p105-Rb"[All Fields] OR "ppp1r130"[All Fields] OR "p110-RB1"[All Fields]) AND ("mouth"[MeSH Terms] OR "mouth"[All Fields] OR "oral"[All Fields]) AND ("carcinoma, squamous cell"[MeSH Terms] OR ("carcinoma"[All Fields] AND "squamous"[All Fields] AND "cell"[All Fields]) OR "squamous cell carcinoma"[All Fields] OR "Neoplasms"[Mesh Terms] OR neopla*[All Fields] OR "cancer"[All Fields])	694	February, 2022
Embase	('retinoblastoma'/exp OR 'rb' OR 'prb' OR 'osrc' OR 'pp110' OR 'p105-Rb' OR 'ppp1r130' OR 'p110-rb1') AND ('mouth'/exp OR 'mouth' OR 'oral') AND ('squamous cell carcinoma'/exp OR 'carcinoma' OR 'malignant neoplasm'/exp OR 'neoplas*' OR 'cancer')	1673	February, 2022
Web of Science	TS=("retinoblastoma" OR "rb" OR "prb" OR "osrc" OR "pp110" OR "p105-Rb" OR "ppp1r130" OR "p110-rb12") AND TS=("mouth" OR "oral") AND TS=("squamous cell carcinoma" OR neoplas* or "cancer")	402	February, 2022
Scopus	TITLE-ABS-KEY(("retinoblastoma" OR "rb" OR "prb" OR "osrc" OR "pp110" OR "p105-Rb" OR "ppp1r130" OR "p110-rb12") AND ("mouth" OR "oral") AND ("squamous cell carcinoma" OR neoplas* or "cancer"))	659	February, 2022
Total			3428

2. Table S2. Characteristics of analyzed studies (n = 20).

Study	Year	Country	Publ. language	Study design	recruitment period	Follow up, m,mean±SD (range)	Sample Size, n	Sex M, n (%) F, n (%)	age, y mean±SD (range)	tumour sites	Tobacco, n (%)	Alcohol, n (%)	Methods	Anti-pRb antibody (clonality)	dilution, incubation time, temperature	IHQ pattern	IHQ cutoff point (%)	Loss pRb (%)
Kühn et al.	2021	Germany	English	Retrospective	NR	<200	78	NR	NR	Oropharynx	NR	NR	IHQ	Ab181616 (monoclonal)	1:700, 1h, Room tª	NR	0	60 (76.92)
Mena et al.	2018	Spain	English	Retrospective	1990-2013	60	784	M: 702 (89.20) F: 85 (10.80)	60.5 ±10.5 NR	Tonsil; posterior tongue; Others	655	603	IHQ	NR	NR	NR	25	502 (64.03)
Vallonthaiel et al.	2016	India	English	Retrospective	2011-2012	Median= 8.5 (1-28)	60	M: 46 (76.67) F: 14 (23.33)	45.6 ± NR (23-72)	Buccal mucosa; Tongue	NR	NR	IHQ	NR (polyclonal)	1:100, Overnight, 4º	Nuclear	30	11 (18.33)
Thomas et al.	2015	India	English	Retrospective	NR	NR	29	NR	NR NR	NR	NR	NR	IHQ	NR (NR)	NR, Overnight, 4ºC,	Nuclear	10	9 (31.03)
Shah et al.	2009	India	English	Retrospective	2000-2003	NR (24-39.14)	135	M:101 (74.82) F: 34 (25.18)	median=45 (28-75)	Buccal mucosa; Tongue	110 (81.48)	NR	IHQ	NR (monoclonal)	1:40, Overnight, 4ºC	Nuclear	10	94 (69.63)
Muirhead et al.	2006	USA	English	Retrospective	NR	<60	45	M: 32 (71.11) F :13(28.89)	Median=58 (25-84)	Tongue; fom; gingiva; rmt; buccal mucosa	NR	NR	IHQ	NR NR	1:25, 30 min, room tª,	Nuclear	0	6 (13.33)
Jayasurya et al.	2005	India	English	Retrospective	NR	Median:23 (1-48)	340	M :219 (62.93) F :129 (37.07)	(60±10)	Tongue; others	31 (9.12)	NR	IHQ	Clone IF8	NR, Overnight, 4ºC	Nuclear	Labelling index (intensity x cell count)	169 (49.71)
Soni et al.	2005	India	English	Retrospective	1993-1999	21-94	220	M: 167 (76) F: 53 (24)	20-85 (59)	Buccal mucosa: gingiva; tongue; lip; others	NR	NR	IHQ	IF8	1:100, NR	Nuclear	10	108 (49)
Liu et al.	2004	Taiwan	English	Retrospective	1985-1996	4-147	55	NR	49.6 (31-64)	NR	NR	NR	IHQ	Clone G3-245, (monoclonal)	1:150, NR	Nuclear, membrane	10	17 (30.90)
Chen et al.	2004	China	Asia	Retrospective	NR	81.89 (6-135)	9	M: 7 (77.78) F: 2 (22.22)	55.67 (44-78)	Oropharynx	NR	NR	IHQ	NR (monoclonal)	1:500 NR NR	Nuclear	25	6 (66.67)
Li et al.	2003	Australia	English	Retrospective	1987-2000	24	86	NR	Median = 57 (30-81)	Tonsils	NR	NR	IHQ	NR (monoclonal)	NR	Epithelium	20	19 (22.09)
van Heerden et al.	2002	South Africa	English	Retrospective	NR	NR	NR	NR	NR	Tongue; Fom	NR	NR	IHQ	NR (NR)	1:25, NR	Nuclear	10	7 (13.21)
Takes et al.	2002	Netherlands	English	Retrospective	1990-1995	NR	52	NR	NR	Oral cavity	NR	NR	IHQ	1F8	1:100, NR	NR	15	12 (23.07)
Nakahara et al.	2000	Japan	English	Retrospective	1989-1998	NR	78	NR	NR	Oral cavity	NR	NR	IHQ	NR	1:40, NR, NR	Nuclear	5	44 (56.41)
Sano et al.	2000	Japan	English	Retrospective	NR	NR	35	M: 17 (48.65) F:18 (51.35)	67.37 (38-87)	Oral cavity	NR	NR	IHQ	G3-245 (monoclonal)	1:200, 4ºC overnight	Nuclear	0	1 (2.85)
Partridge et al.	1999	England	English	Retrospective	NR	47.36 (28-166)	44	M:27 (48.36) F:17 (38.64)	59.75 (31-88)	Tongue; Buccal mucosa; Gingiva; Fom;	24	16	IHQ	Ab-5 (monoclonal)	1:6 NR NR	Nuclear	20	9 (20.45)
Tanaka et al.	1999	Japan	English	Retrospective	NR	NR	110	M: 84 (76.36) F: 26(23.64)	60.40 (28-89)	Tongue; Gingiva; fom; Oropharynx; Buccal mucosa	NR	NR	IHQ	Clone3H9 (monoclonal)	1:100 Overnight 4ºC	Nuclear	10	27 (24.55)

Pande et al.	1998	India	Asia	Retrospective	NR	47.36 (1-166)	35	M: 31 (88.57)<br>F: 4 (11.43)	53.8 (25-85)	Bucal mucosa;<br>Tongue;<br>Gingiva;<br>Palate	31	NR	IHQ	IF8<br>(monoclonal)	1:100<br>Overnight<br>4ºC	Nuclear	0	23<br>(65.71)
Xu et al.	1997	USA	America	Retrospective	1985-1992	NR	24	M: 18 (85.71)<br>F: 3 (14.29)	62.57 (35-87)	Tongue<br>fom<br>buccal mucosa	21	15	IHQ	Ab-4<br>(monoclonal)	NR<br>Overnight<br>4ºC	Nuclear	5	1<br>(4.76)
Pavelic et al.	1996	USA	America	Retrospective	NR	>17	182	NR	NR	NR	NR	NR	IHQ	LM95.1<br>(monoclonal)	1:10<br>NR<br>NR	Nuclear	0	47<br>(25.82)

Abbreviations: Bm, buccal mucosa; fom, floor of mouth; OSCC, oral squamous cell carcinoma; m, months; n, number; NR, not reported; RoB, risk of bias; SD, standard deviation; y, years; .

3. Meta-analysis on the loss of pRb expression and overall survival in OSCC

3.1 Subgroup meta-analysis by geographical area

Figure S1. Forest plot graphically representing the stratified analysis by geographical area on the association between the loss of pRb expression and overall survival in patients with OSCC.

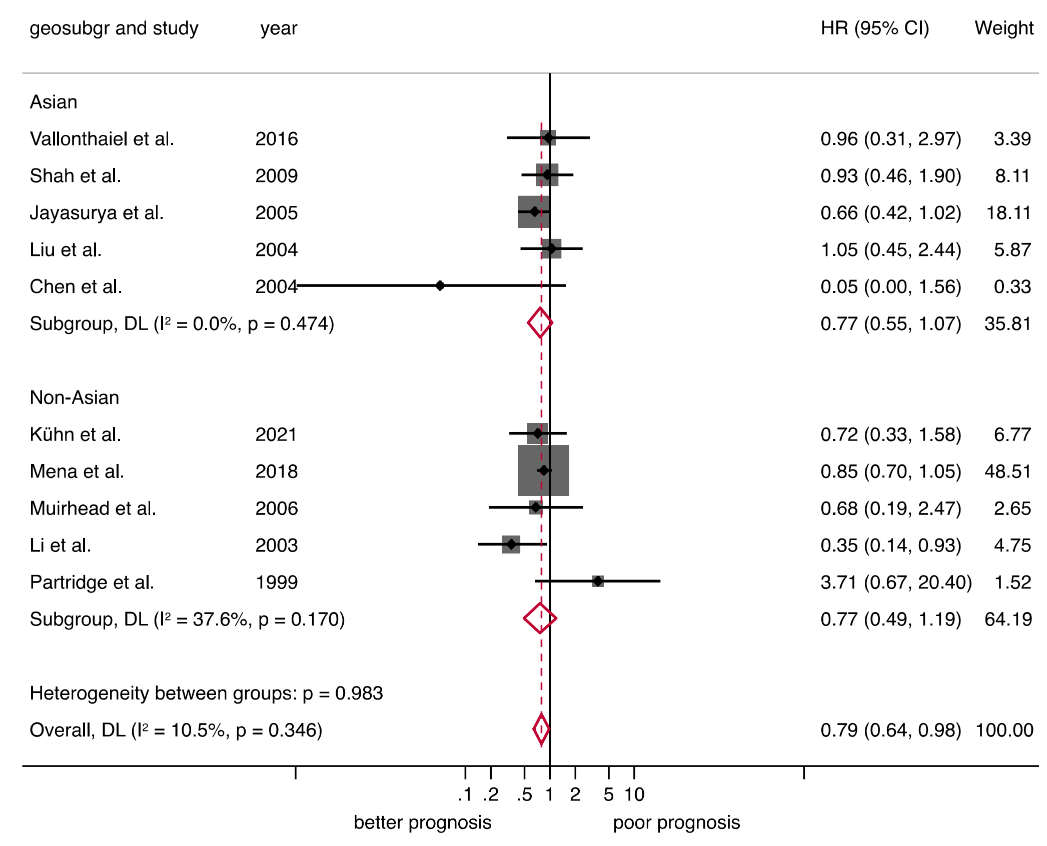

OSCC, oral squamous cell carcinoma; HR, hazard ratio; CI, confidence intervals. Random-effects model, inverse-variance weighting (based on the DerSimonian and Laird method). A HR > 1 suggests that the loss of pRb expression expression is associated with poor overall survival. Diamonds indicate the pooled HRs with their corresponding 95% CIs.

3.2 Subgroup meta-analysis by anti-pRb antibody

Figure S2. Forest plot graphically representing the stratified analysis by anti-pRb antibody on the association between the loss of pRb expression and overall survival in patients with OSCC.

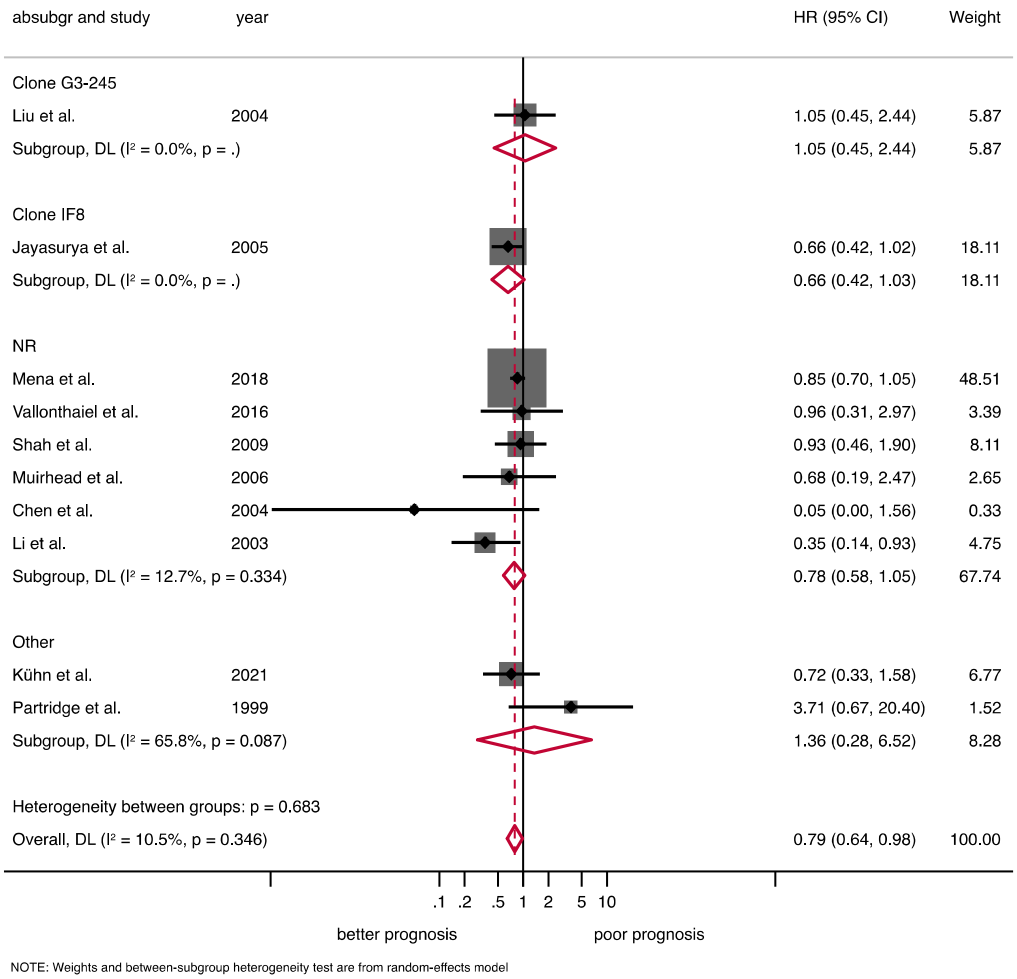

OSCC, oral squamous cell carcinoma; HR, hazard ratio; CI, confidence intervals. Random-effects model, inverse-variance weighting (based on the DerSimonian and Laird method). A HR > 1 suggests that the loss of pRb expression is associated with poor overall survival. Diamonds indicate the pooled HRs with their corresponding 95% CIs.

3.3 Subgroup meta-analysis by anti-pRb antibody dilution

Figure S3. Forest plot graphically representing the stratified analysis by anti-pRb antibody dilution on the association between the loss of pRb expression and overall survival in patients with OSCC.

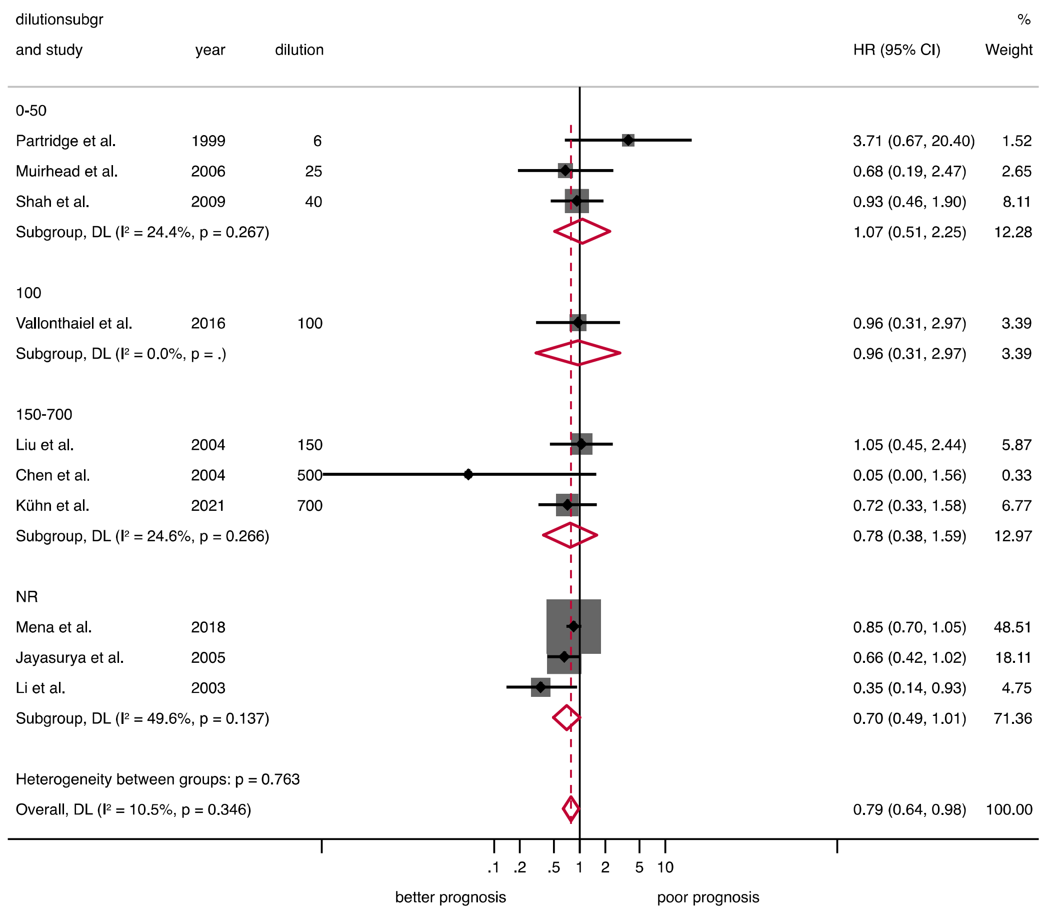

OSCC, oral squamous cell carcinoma; HR, hazard ratio; CI, confidence intervals. Random-effects model, inverse-variance weighting (based on the DerSimonian and Laird method). A HR > 1 suggests that the loss of pRb expression is associated with poor overall survival. Diamonds indicate the pooled HRs with their corresponding 95% CIs.

3.4 Subgroup meta-analysis by anti-pRb antibody incubation time

Figure S4. Forest plot graphically representing the stratified analysis by anti-pRb antibody incubation time on the association between the loss of pRb expression and overall survival in patients with OSCC.

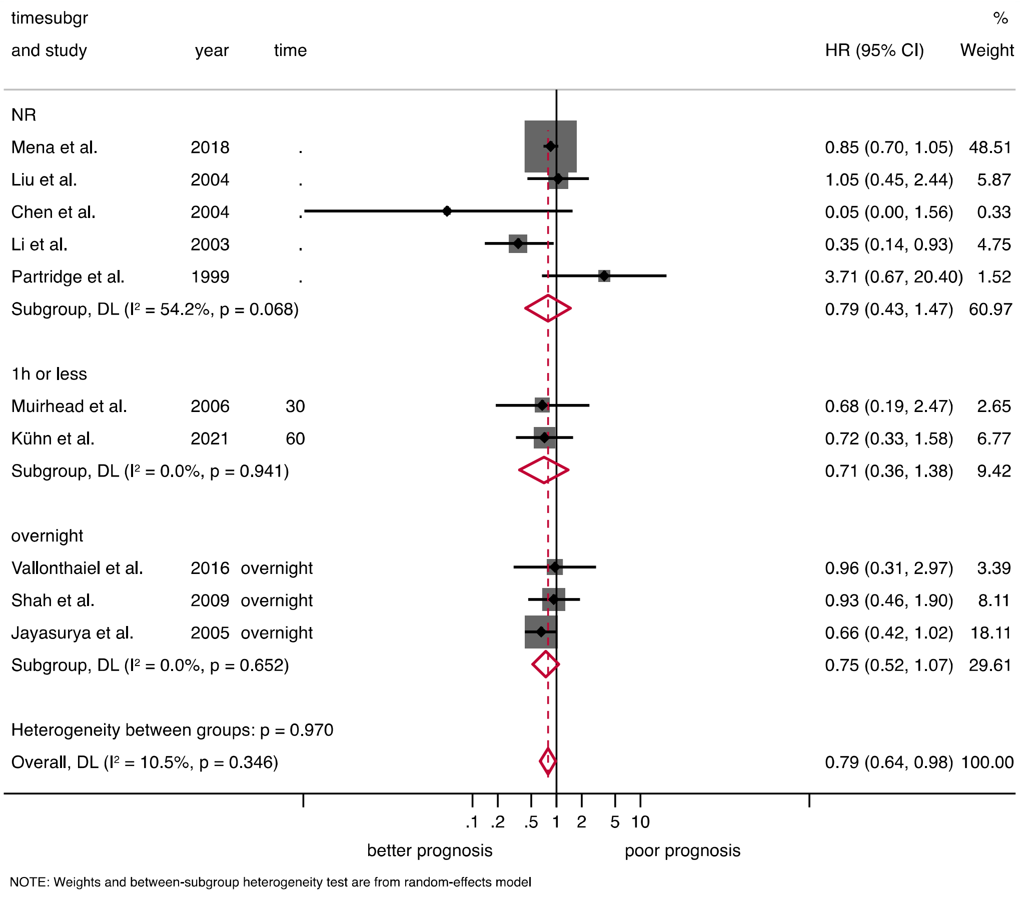

OSCC, oral squamous cell carcinoma; HR, hazard ratio; CI, confidence intervals. Random-effects model, inverse-variance weighting (based on the DerSimonian and Laird method). A HR > 1 suggests that the loss of pRb expression is associated with poor overall survival. Diamonds indicate the pooled HRs with their corresponding 95% CIs.

3.5 Subgroup meta-analysis by anti-pRb antibody incubation temperature

Figure S5. Forest plot graphically representing the stratified analysis by anti-pRb antibody incubation temperature on the association between the loss of pRb expression and overall survival in patients with OSCC.

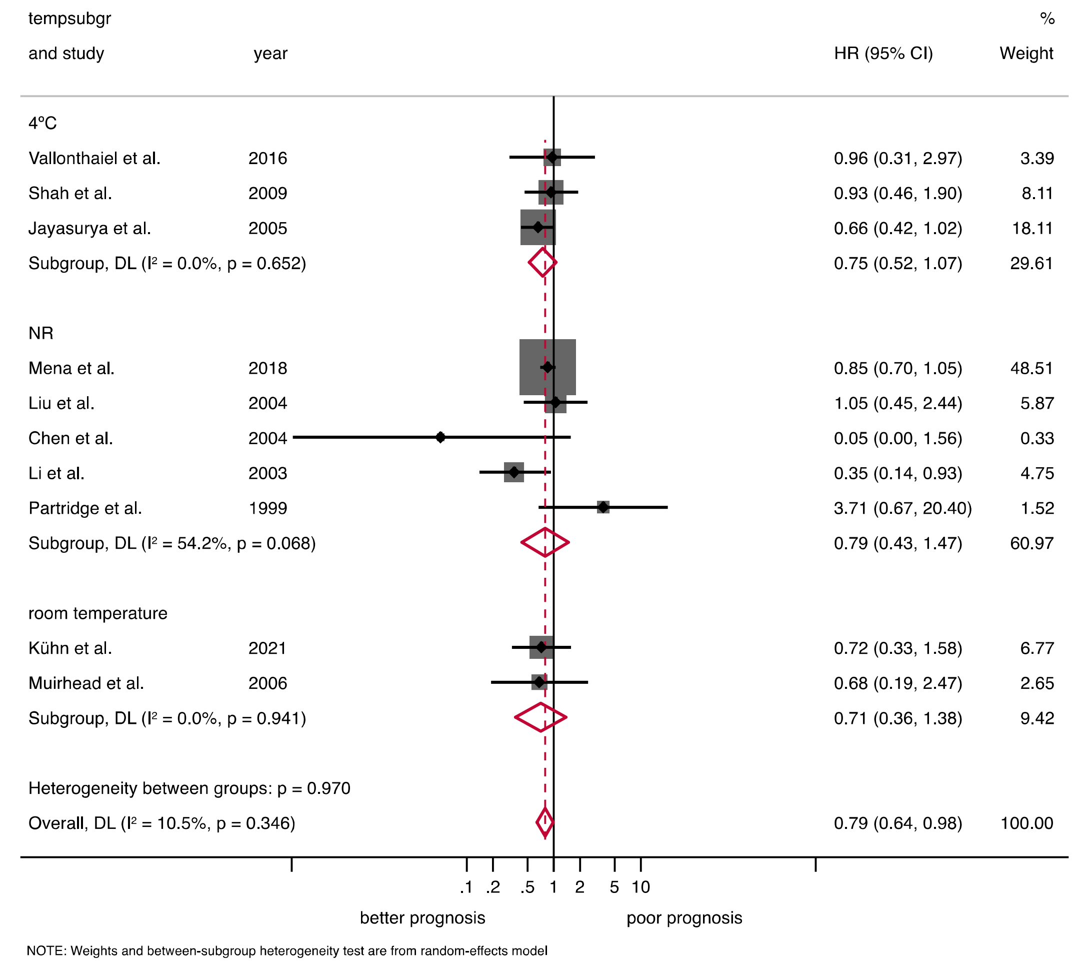

OSCC, oral squamous cell carcinoma; HR, hazard ratio; CI, confidence intervals. Random-effects model, inverse-variance weighting (based on the DerSimonian and Laird method). A HR > 1 suggests that the loss of pRb expression is associated with poor overall survival. Diamonds indicate the pooled HRs with their corresponding 95% CIs.

3.6 Subgroup meta-analysis by cut-off point

Figure S6. Forest plot graphically representing the stratified analysis by cut-off point on the association between the loss of pRb expression and overall survival in patients with OSCC.

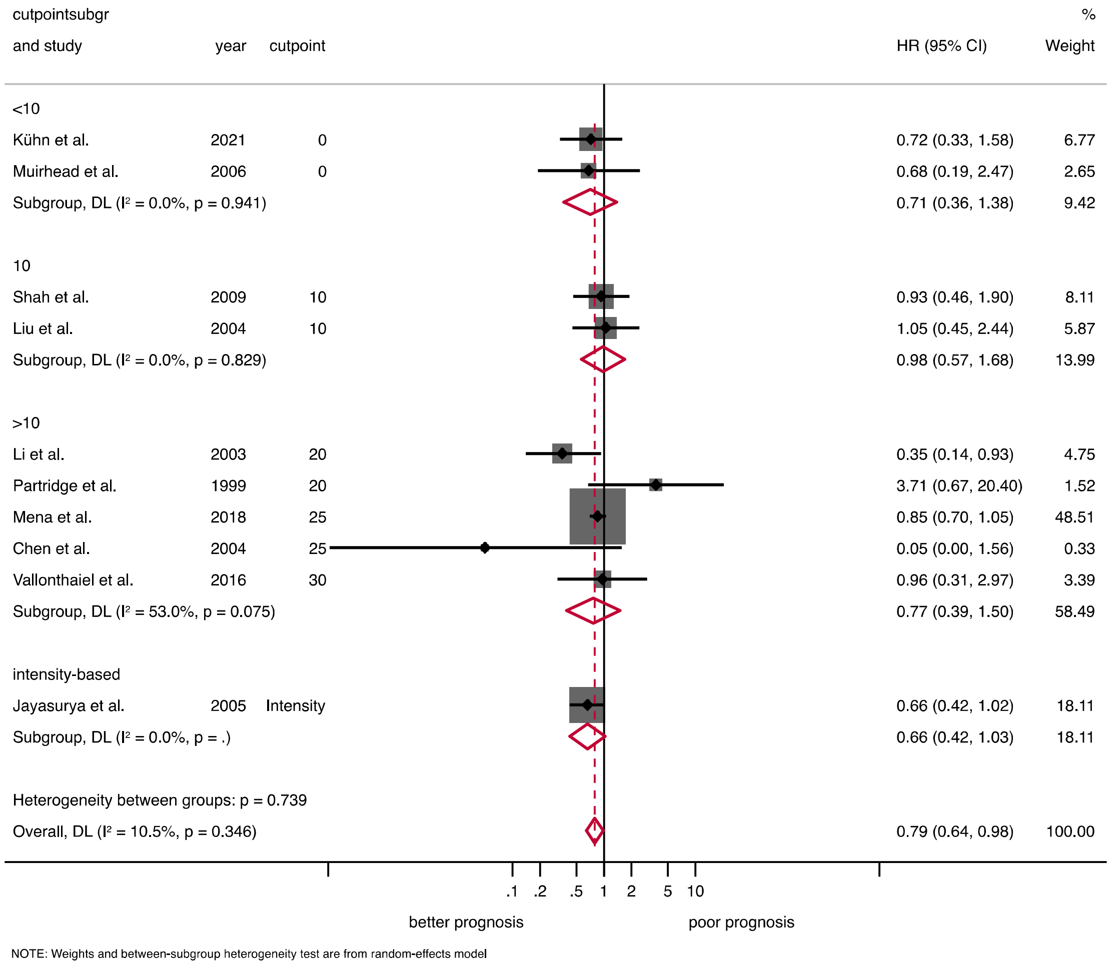

OSCC, oral squamous cell carcinoma; HR, hazard ratio; CI, confidence intervals. Random-effects model, inverse-variance weighting (based on the DerSimonian and Laird method). A HR > 1 suggests that the loss of pRb expression is associated with poor overall survival. Diamonds indicate the pooled HRs with their corresponding 95% CIs.

3.7 Subgroup meta-analysis by overall risk of bias in primary-level studies

Figure S7. Forest plot graphically representing the stratified analysis by overall RoB in primary-level studies, on the association between the loss of pRb expression and overall survival in patients with OSCC.

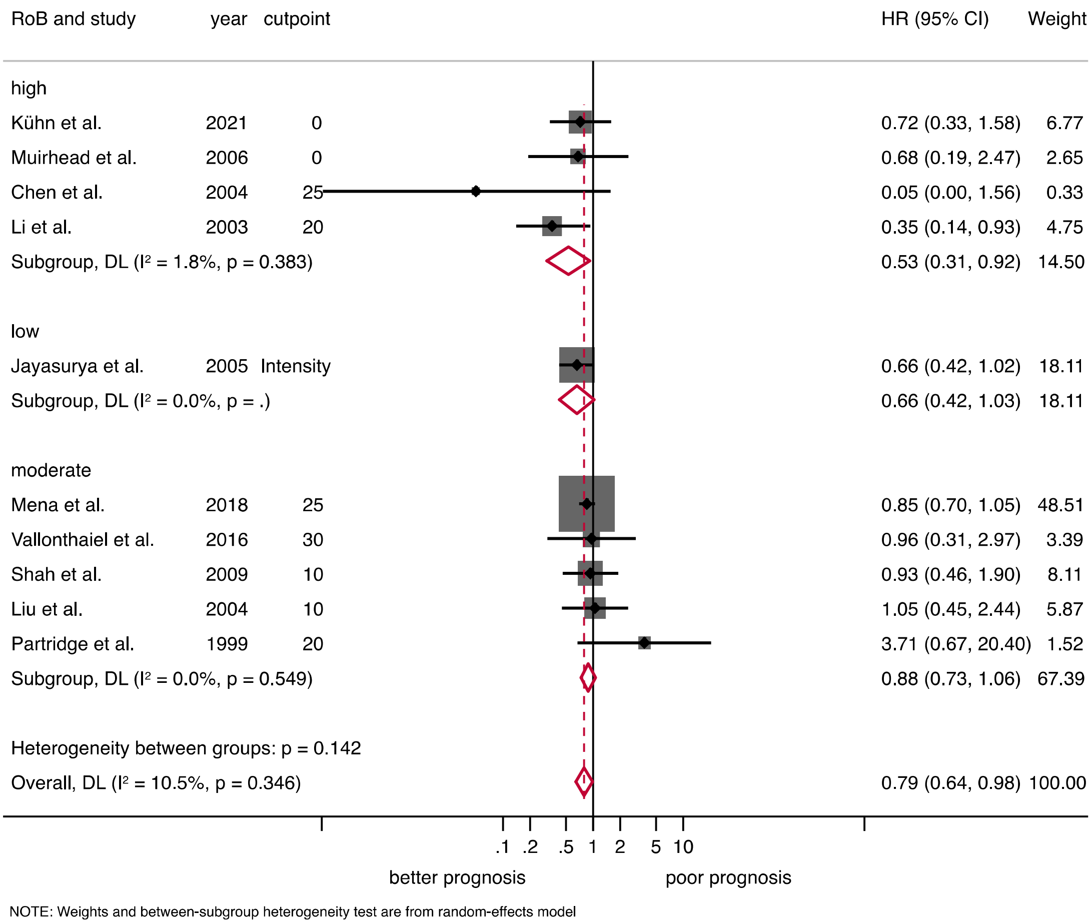

OSCC, oral squamous cell carcinoma; RoB, risk of bias; HR, hazard ratio; CI, confidence intervals. Random-effects model, inverse-variance weighting (based on the DerSimonian and Laird method). A HR > 1 suggests that the loss of pRb expression is associated with poor overall survival. Diamonds indicate the pooled HRs with their corresponding 95% CIs.

3.8 Univariable meta-regression on the effect of follow up

Figure S8. Bubble plot graphically representing the univariable meta-regression analysis of the potential effect of follow up period (expressed in months, in x-axis) on the association between the loss of pRb expression and overall survival in patients with OSCC (using HR as effect size measure, in y-axis).

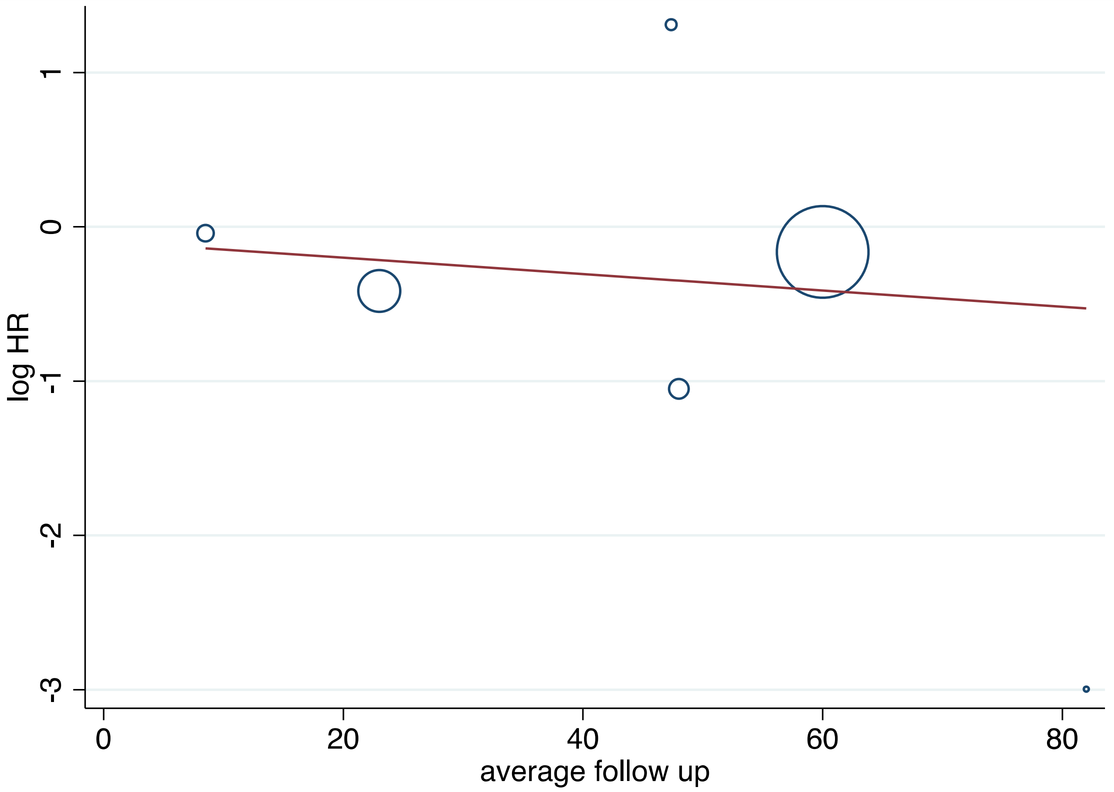

OSCC, oral squamous cell carcinoma; HR, hazard ratio; log, natural logarithm (i.e., log base e). The red line exhibits the fitted regression line together with blue circles representing the estimates from each individual study, sized according to the precision of each estimate (the inverse of its within-study variance).

3.9 Univariable meta-regression on the effect of sex

Figure S9. Bubble plot graphically representing the univariable meta-regression analysis of the potential effect of sex (% of males) on the association between the loss of pRb expression and overall survival in patients with OSCC.

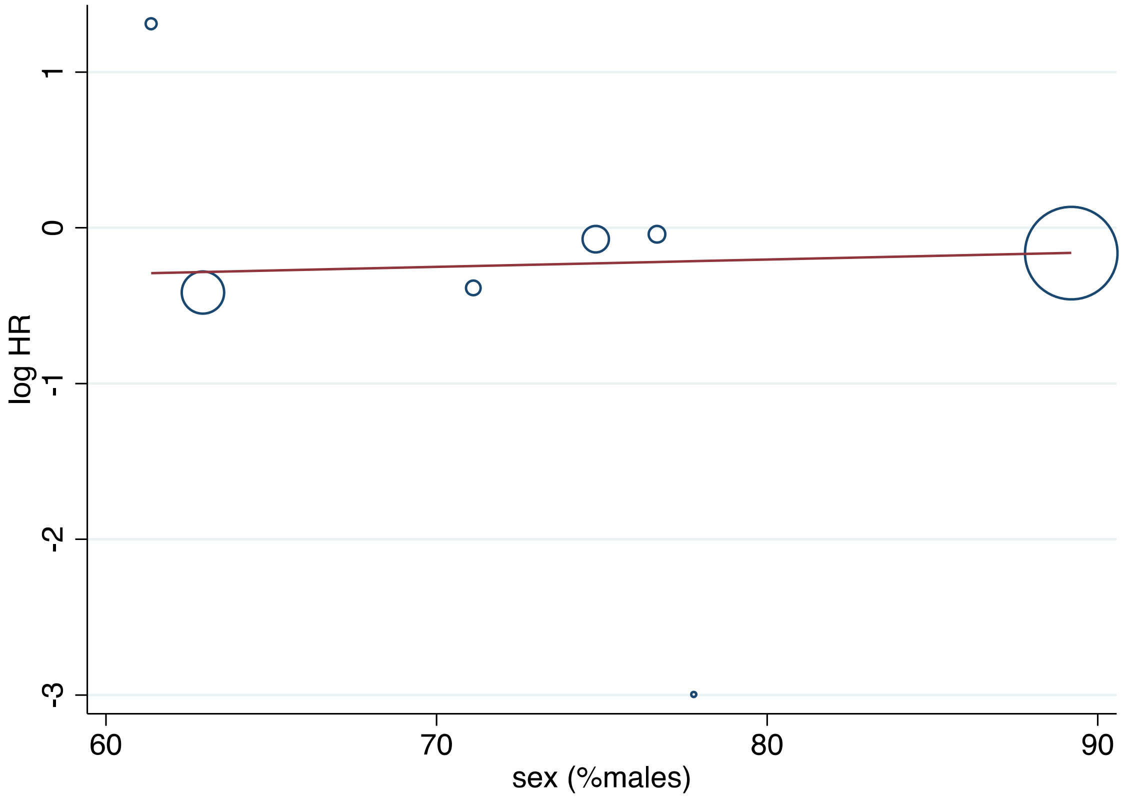

OSCC, oral squamous cell carcinoma; HR, hazard ratio; log, natural logarithm (i.e., log base e). The red line exhibits the fitted regression line together with blue circles representing the estimates from each individual study, sized according to the precision of each estimate (the inverse of its within-study variance).

3.10 Univariable meta-regression on the effect of age

Figure S10. Bubble plot graphically representing the univariable meta-regression analysis of the potential effect of age (mean age of patients, expressed in years) on the association between the loss of pRb expression and overall survival in patients with OSCC.

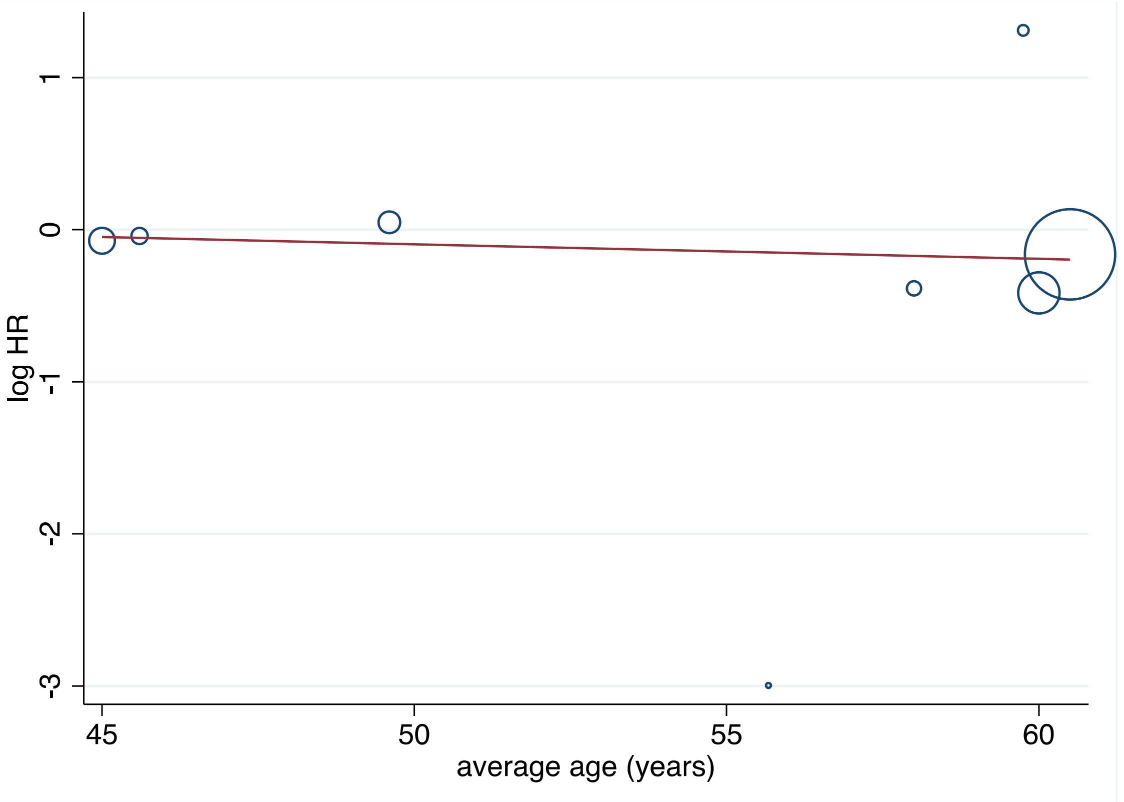

OSCC, oral squamous cell carcinoma; HR, hazard ratio; log, natural logarithm (i.e., log base e). The red line exhibits the fitted regression line together with blue circles representing the estimates from each individual study, sized according to the precision of each estimate (the inverse of its within-study variance).

4. Meta-analysis on the loss of pRb expression and DFS in OSCC

Figure S11. Forest plot graphically representing the meta-analysis on the association between the loss of pRb expression and DFS in patients with OSCC.

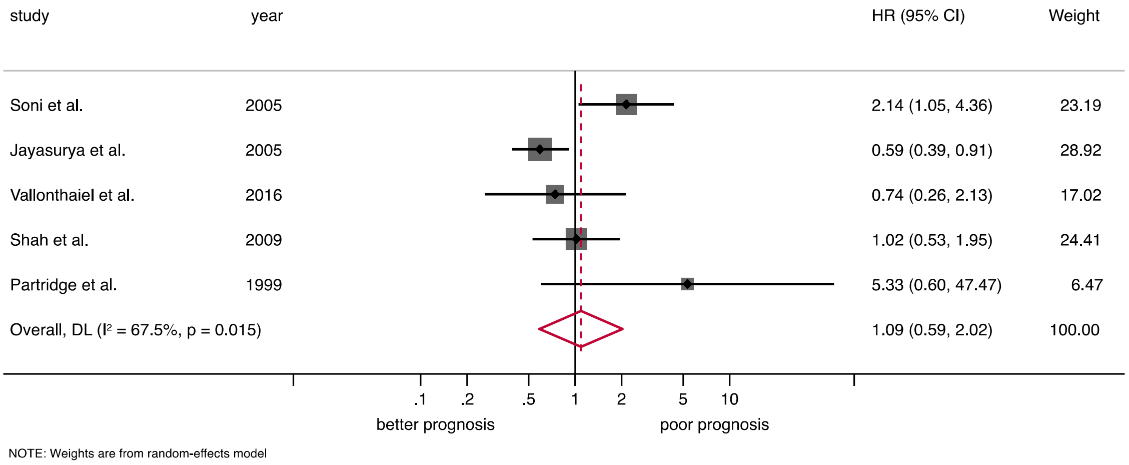

DFS, disease-free survival; OSCC, oral squamous cell carcinoma; HR, hazard ratio; CI, confidence intervals. Random-effects model, inverse-variance weighting (based on the DerSimonian and Laird method). A HR > 1 suggests that the loss of pRb expression is associated with poor DFS. Diamonds indicate the pooled HRs with their corresponding 95% CIs.

5. Meta-analysis on the loss of pRb expression and T status in OSCC

Figure S12. Forest plot graphically representing the meta-analysis on the association between the loss of pRb expression and T status (T3/T4 vs. T1/T2) in patients with OSCC.

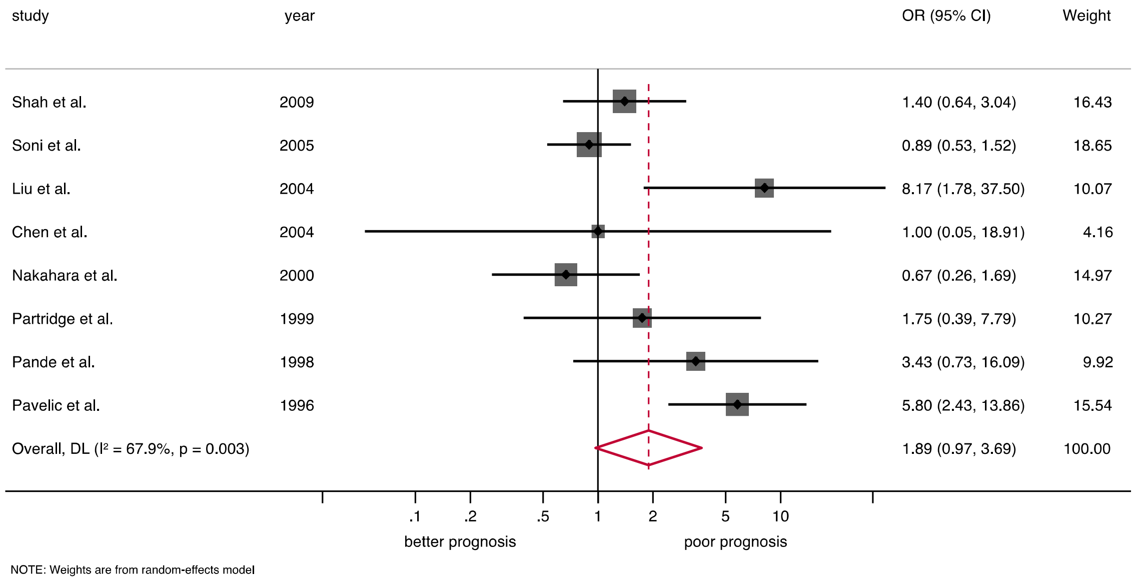

OSCC, oral squamous cell carcinoma; OR, odds ratio; CI, confidence intervals. Random-effects model, inverse-variance weighting (based on the DerSimonian and Laird method). A OR > 1 suggests that the loss of pRb expression is associated with a higher T status. Diamonds indicate the pooled ORs with their corresponding 95% CIs.

6. Meta-analysis on the loss of pRb expression and N status in OSCC

Figure S13. Forest plot graphically representing the meta-analysis on the association between the loss of pRb expression and N status (positive metastatic lymph nodes vs. negative) in patients with OSCC.

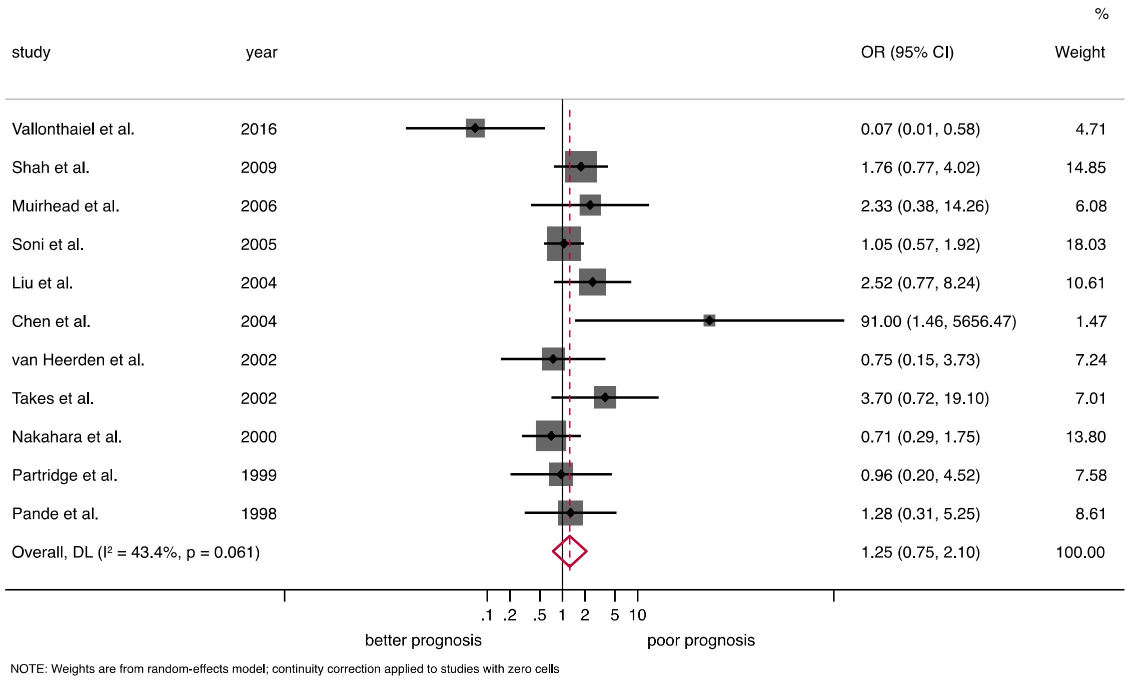

OSCC, oral squamous cell carcinoma; OR, odds ratio; CI, confidence intervals. Random-effects model, inverse-variance weighting (based on the DerSimonian and Laird method). A OR > 1 suggests that the loss of pRb expression is associated with positive N status. Diamonds indicate the pooled ORs with their corresponding 95% CIs.

7. Meta-analysis on the loss of pRb expression and clinical stage in OSCC

Figure S14. Forest plot graphically representing the meta-analysis on the association between the loss of pRb expression and clinical stage (III/IV vs. I/II) in patients with OSCC.

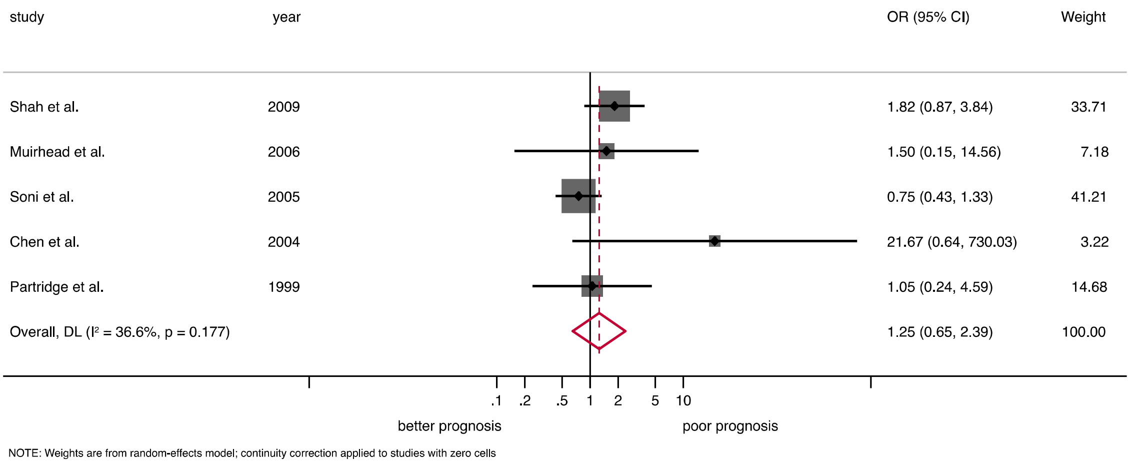

OSCC, oral squamous cell carcinoma; OR, odds ratio; CI, confidence intervals. Random-effects model, inverse-variance weighting (based on the DerSimonian and Laird method). A OR > 1 suggests that the loss of pRb expression is associated with a higher stage. Diamonds indicate the pooled ORs with their corresponding 95% CIs.

8. Meta-analysis on the loss of pRb expression and histological grade in OSCC

Figure S15. Forest plot graphically representing the meta-analysis on the association between the loss of pRb expression and histological grade (poorly-moderate vs. well-differentiated carcinomas) in patients with OSCC.

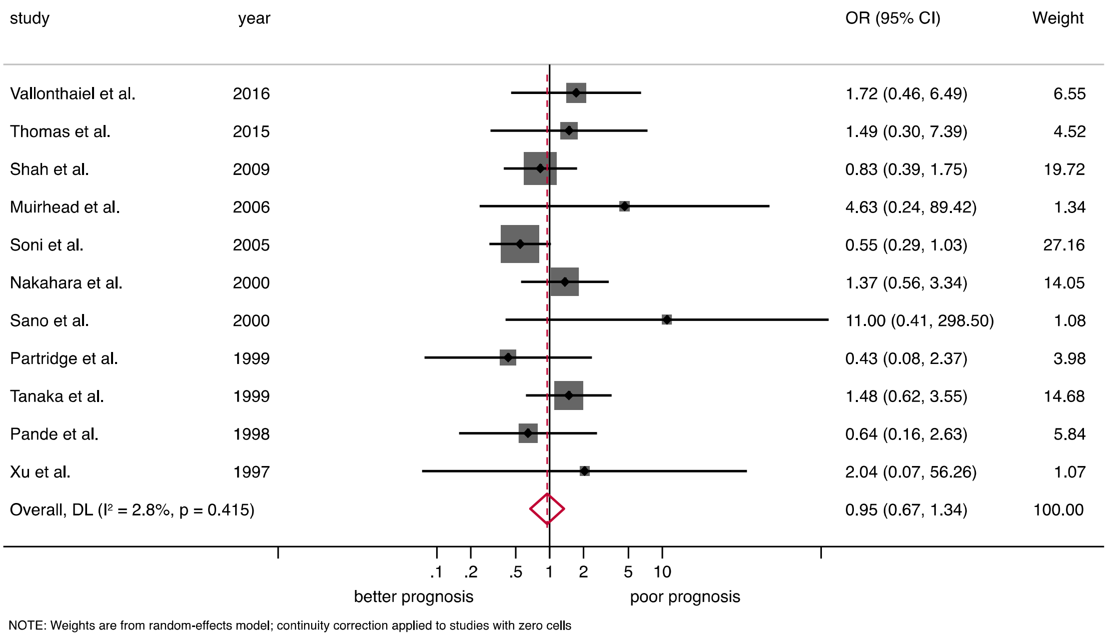

OSCC, oral squamous cell carcinoma; OR, odds ratio; CI, confidence intervals. Random-effects model, inverse-variance weighting (based on the DerSimonian and Laird method). A OR > 1 suggests that the loss of pRb expression is associated with a higher grade. Diamonds indicate the pooled ORs with their corresponding 95% CIs.

9. Analysis of small-study effects

9.1 The loss of pRb expression and overall survival in OSCC

Figure S16. A funnel plot of estimated logHRs against their standard errors, graphically representing the analysis of small-study effects on the association between the loss of pRb expression and overall survival in OSCC.

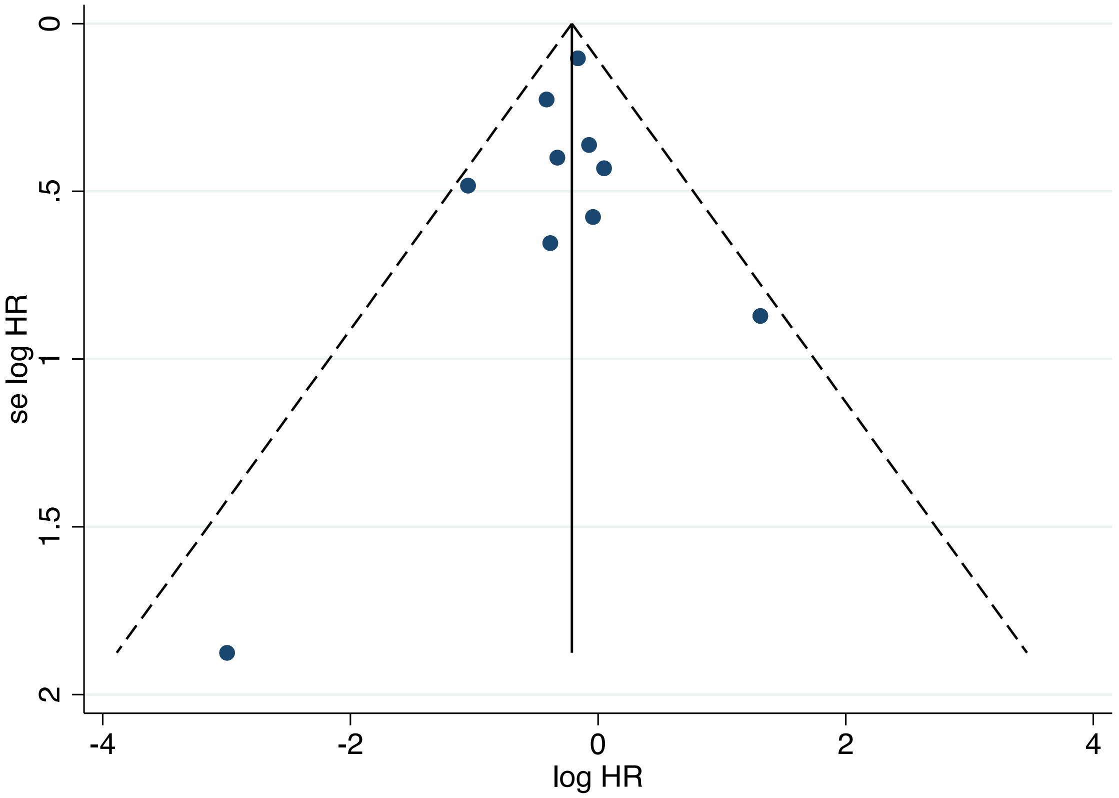

SE, standard error; HR, hazard ratio; log, natural logarithm (i.e., log base e). The black vertical line corresponds to the pooled estimated prevalence. The two diagonal intermittent lines represent the pseudo-95% confidence interval. The blue circles represent the estimates from primary-level studies.

9.2 The loss of pRb expression and DFS in OSCC

Figure S17. A funnel plot of estimated logHRs against their standard errors, graphically representing the analysis of small-study effects on the association between the loss of pRb expression and DFS in OSCC.

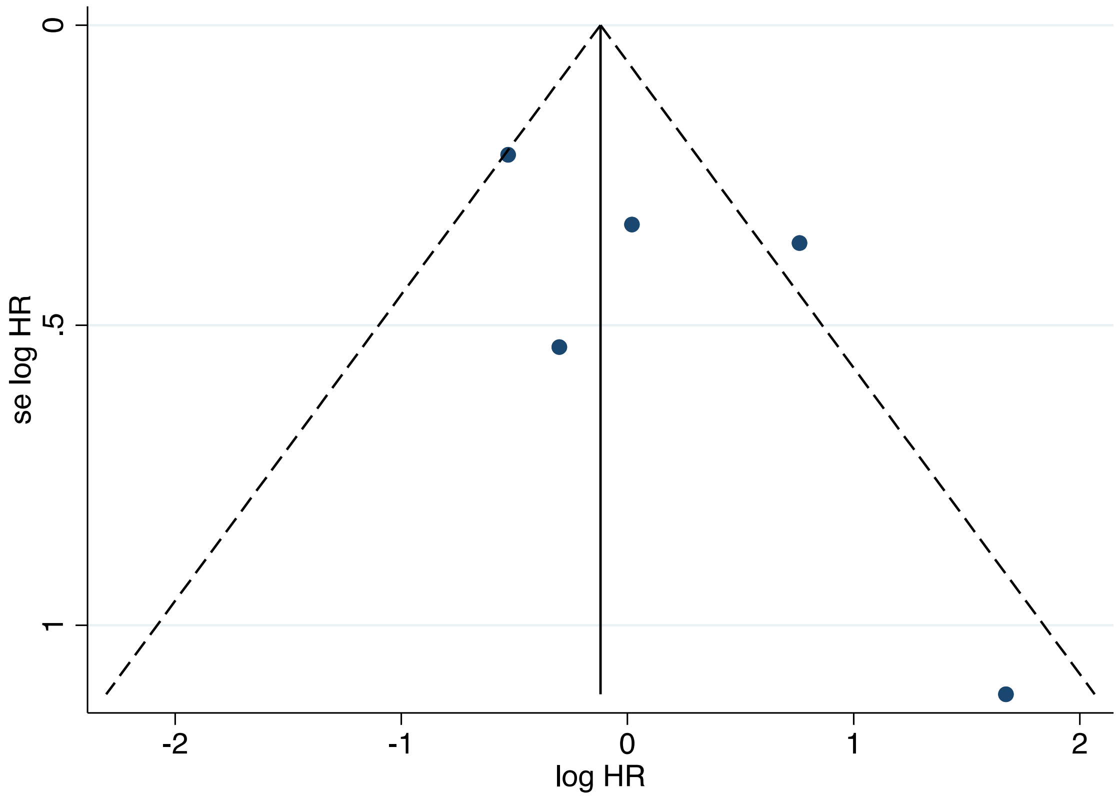

DFS, disease-free survival; SE, standard error; HR, hazard ratio; log, natural logarithm (i.e., log base e). The black vertical line corresponds to the pooled estimated prevalence. The two diagonal intermittent lines represent the pseudo-95% confidence interval. The blue circles represent the estimates from primary-level studies.

9.3 The loss of pRb expression and T status in OSCC

Figure S18. A funnel plot of estimated logORs against their standard errors, graphically representing the analysis of small-study effects on the association between the loss of pRb expression and T status in OSCC.

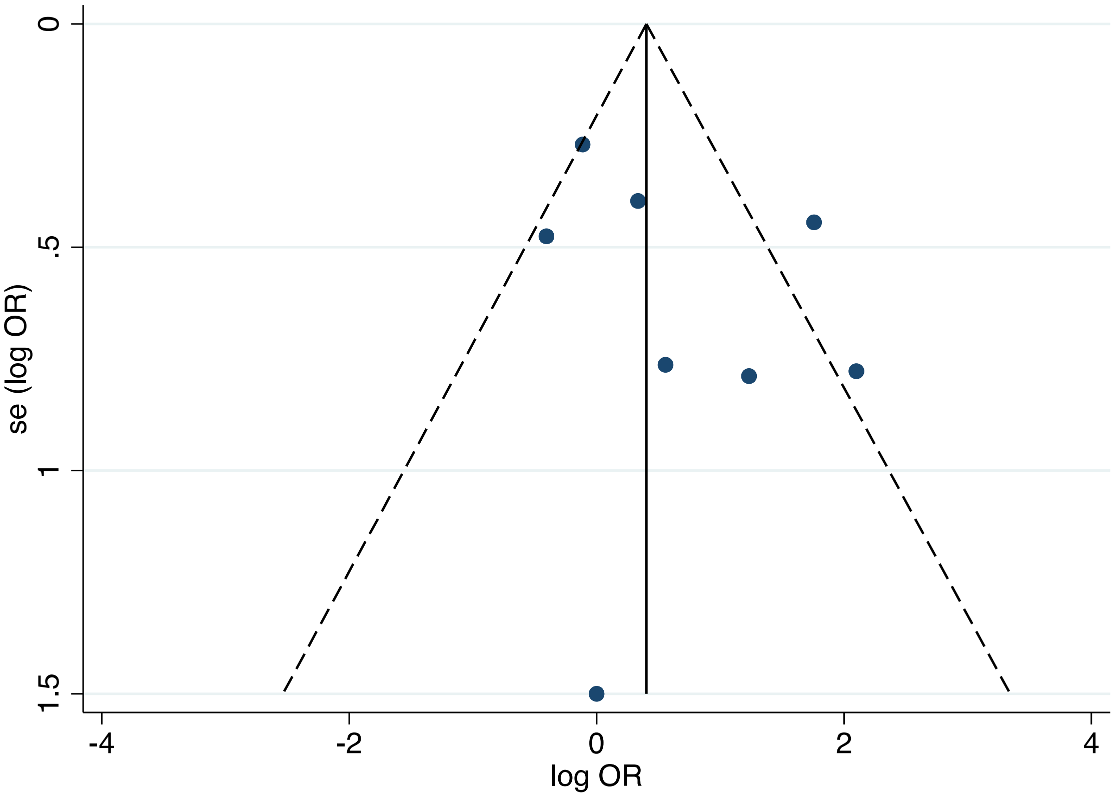

SE, standard error; OR, odds ratio; log, natural logarithm (i.e., log base e). The black vertical line corresponds to the pooled estimated prevalence. The two diagonal intermittent lines represent the pseudo-95% confidence interval. The blue circles represent the estimates from primary-level studies.

9.4 The loss of pRb expression and N status in OSCC

Figure S19. A funnel plot of estimated logORs against their standard errors, graphically representing the analysis of small-study effects on the association between the loss of pRb expression and N status in OSCC.

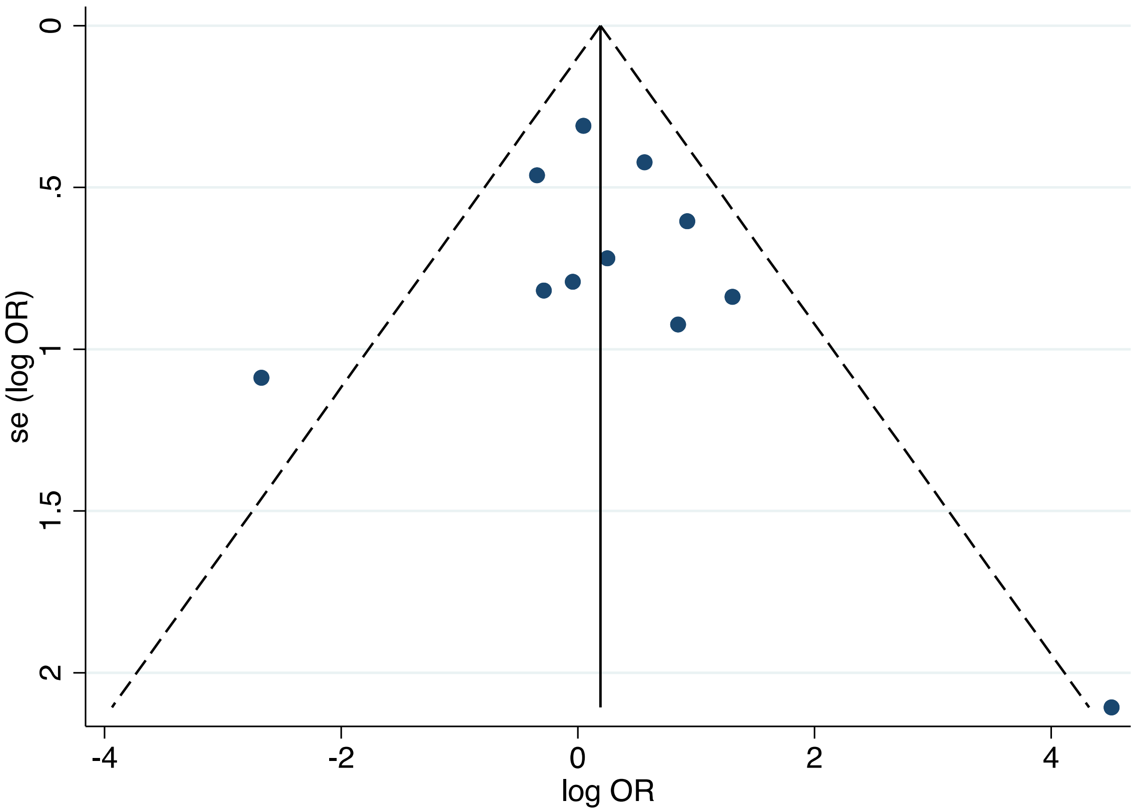

SE, standard error; OR, odds ratio; log, natural logarithm (i.e., log base e). The black vertical line corresponds to the pooled estimated prevalence. The two diagonal intermittent lines represent the pseudo-95% confidence interval. The blue circles represent the estimates from primary-level studies.

9.5 The loss of pRb expression and clinical stage in OSCC

Figure S20. A funnel plot of estimated logORs against their standard errors, graphically representing the analysis of small-study effects on the association between the loss of pRb expression and clinical stage in OSCC.

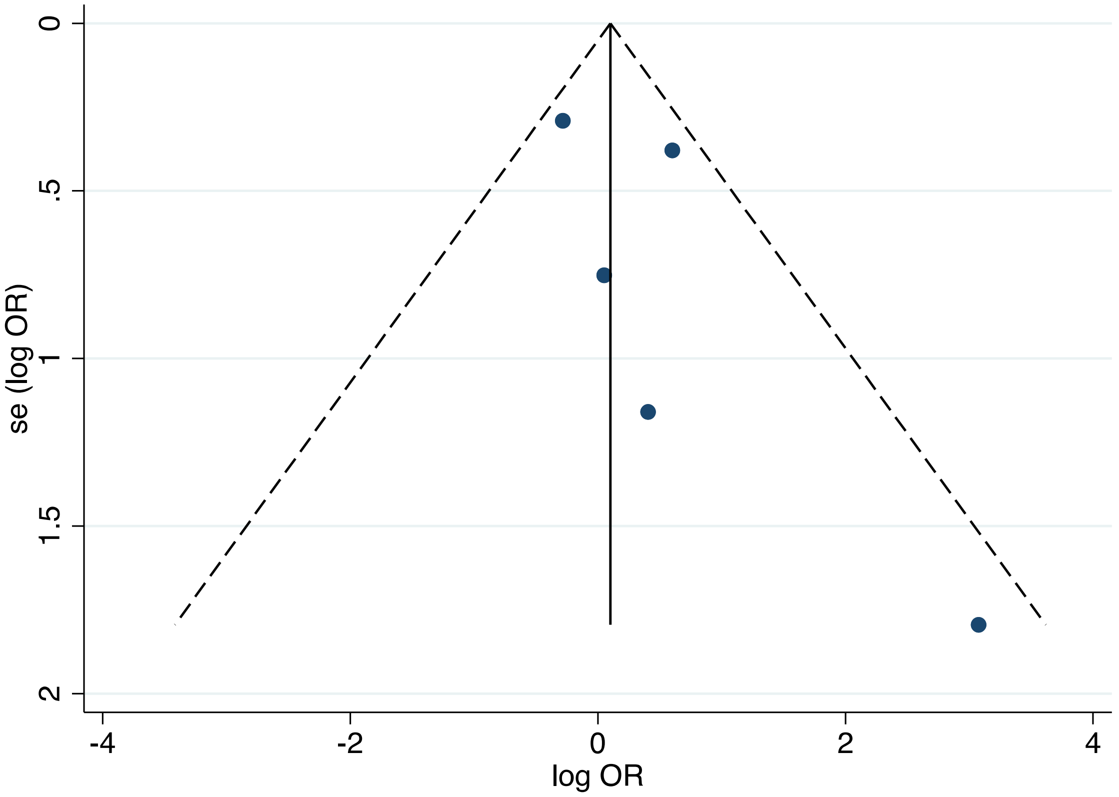

SE, standard error; OR, odds ratio; log, natural logarithm (i.e., log base e). The black vertical line corresponds to the pooled estimated prevalence. The two diagonal intermittent lines represent the pseudo-95% confidence interval. The blue circles represent the estimates from primary-level studies.

9.6 The loss of pRb expression and histological grade in OSCC

Figure S21. A funnel plot of estimated logORs against their standard errors, graphically representing the analysis of small-study effects on the association between the loss of pRb expression and histological grade in OSCC.

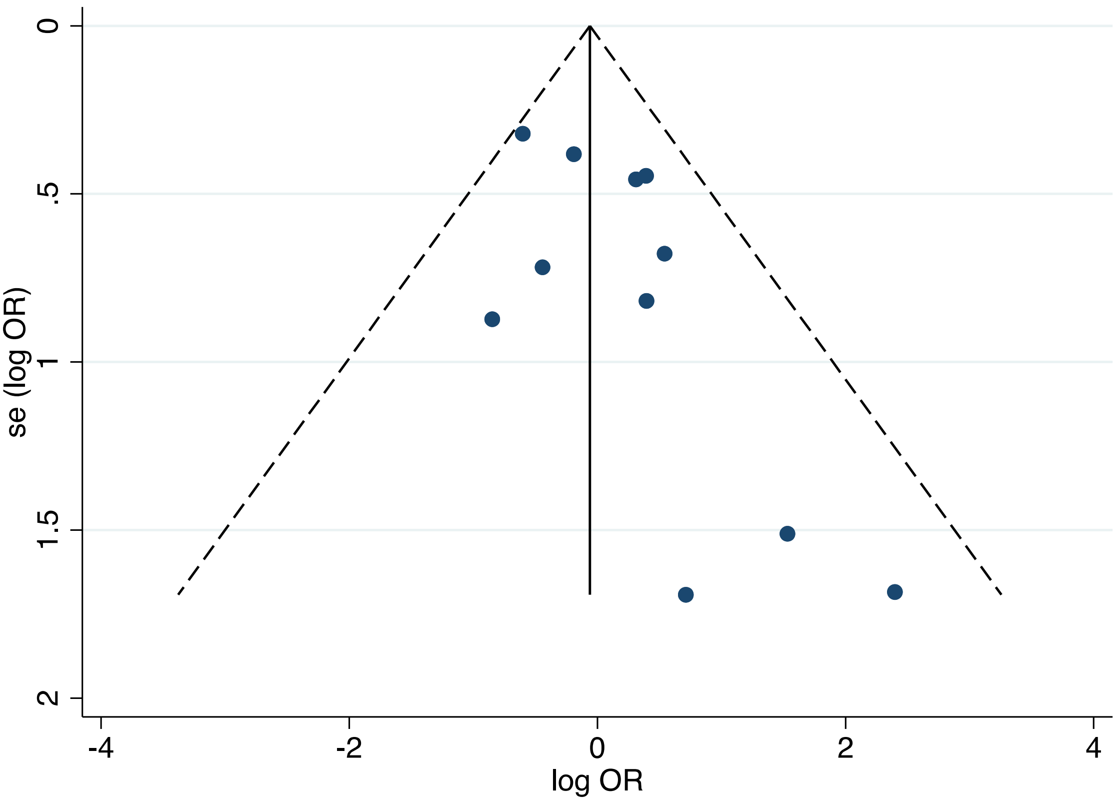

SE, standard error; OR, odds ratio; log, natural logarithm (i.e., log base e). The black vertical line corresponds to the pooled estimated prevalence. The two diagonal intermittent lines represent the pseudo-95% confidence interval. The blue circles represent the estimates from primary-level studies.

10. List S1: List of full-text articles excluded with reasons

<u>No clinico-pathological outcomes (n=18)</u>

1. Antonioli, M., Pagni, B., Vescovo, T., Ellis, R., Cosway, B., Rollo, F., Bordoni, V., Agrati, C., Labus, M., Covello, R., Benevolo, M., Ippolito, G., Robinson, M., Piacentini, M., Lovat, P., & Fimia, G. M. (2021). HPV sensitizes OPSCC cells to cisplatin-induced apoptosis by inhibiting autophagy through E7-mediated degradation of AMBRA1. *Autophagy*, *17*(10), 2842–2855.
2. Shaikh, M. H., Khan, A. I., Sadat, A., Chowdhury, A. H., Jinnah, S. A., Gopalan, V., Lam, A. K., Clarke, D., McMillan, N., & Johnson, N. W. (2017). Prevalence and types of high-risk human papillomaviruses in head and neck cancers from Bangladesh. *BMC cancer*, *17*(1), 792.
3. Castellsagué, X., Alemany, L., Quer, M., Halec, G., Quirós, B., Tous, S., Clavero, O., Alòs, L., Biegner, T., Szafarowski, T., Alejo, M., Holzinger, D., Cadena, E., Claros, E., Hall, G., Laco, J., Poljak, M., Benevolo, M., Kasamatsu, E., Mehanna, H., … ICO International HPV in Head and Neck Cancer Study Group (2016). HPV Involvement in Head and Neck Cancers: Comprehensive Assessment of Biomarkers in 3680 Patients. *Journal of the National Cancer Institute*, *108*(6), djv403.
4. Bascones-Martínez, A., López-Durán, M., Cano-Sánchez, J., Sánchez-Verde, L., Díez-Rodríguez, A., Aguirre-Echebarría, P., Alvarez-Fernández, E., González-Moles, M. A., Bascones-Ilundain, J., Muzio, L. L., & Campo-Trapero, J. (2012). Differences in the expression of five senescence markers in oral cancer, oral leukoplakia and control samples in humans. *Oncology letters*, *3*(6), 1319–1325.
5. Nasser, W., Flechtenmacher, C., Holzinger, D., Hofele, C., & Bosch, F. X. (2011). Aberrant expression of p53, p16INK4a and Ki-67 as basic biomarker for malignant progression of oral leukoplakias. *Journal of oral pathology & medicine : official publication of the International Association of Oral Pathologists and the American Academy of Oral Pathology*, *40*(8), 629–635.
6. Park, J. M., Jung, C. K., Choi, Y. J., Lee, K. Y., Kang, J. H., Kim, M. S., & Hu, H. J. (2010). The use of an immunohistochemical diagnostic panel to determine the primary site of cervical lymph node metastases of occult squamous cell carcinoma. *Human pathology*, *41*(3), 431–437.
7. Soares, R. C., Oliveira, M. C., de Souza, L. B., Costa, A., & Pinto, L. P. (2008). Detection of HPV DNA and immunohistochemical expression of cell cycle proteins in oral carcinoma in a population of Brazilian patients. *Journal of applied oral science : revista FOB*, *16*(5), 340–344.
8. Sathyan, K. M., Nalinakumari, K. R., & Kannan, S. (2007). H-Ras mutation modulates the expression of major cell cycle regulatory proteins and disease prognosis in oral carcinoma. *Modern pathology : an official journal of the United States and Canadian Academy of Pathology, Inc*, *20*(11), 1141–1148.
9. Nemes, J. A., Deli, L., Nemes, Z., & Márton, I. J. (2006). Expression of p16(INK4A), p53, and Rb proteins are independent from the presence of human papillomavirus genes in oral squamous cell carcinoma. *Oral surgery, oral medicine, oral pathology, oral radiology, and endodontics*, *102*(3), 344–352.
10. Hofele, C., Joos, S., Flechtenmacher, C., Bosch, F. X., Lichter, P., Mühling, J., & Freier, K. (2002). Möglichkeiten und chancen der Gewebechiptechnologie bei Kopf-Hals-Tumoren. Eine neue Technik zur schnellen Analyse von potenziellen Tumormarkern [Opportunities and chances for tissue chip microarrays in head and neck surgery. A novel technique for the rapid evaluation of potentially novel biomarkers]. *Mund-, Kiefer- und Gesichtschirurgie : MKG*, *6*(6), 394–401.

11. Haas, S., Hörmann, K., & Bosch, F. X. (2002). Expression of cell cycle proteins in head and neck cancer correlates with tumor site rather than tobacco use. *Oral oncology*, *38*(6), 618–623.
12. Koontongkaew, S., Chareonkitkajorn, L., Chanvitan, A., Leelakriangsak, M., & Amornphimoltham, P. (2000). Alterations of p53, pRb, cyclin D(1) and cdk4 in human oral and pharyngeal squamous cell carcinomas. *Oral oncology*, *36*(4), 334–339.
13. Regezi, J. A., Dekker, N. P., McMillan, A., Ramirez-Amador, V., Meneses-Garcia, A., Ruiz-Godoy Rivera, L. M., Chrysomali, E., & Ng, I. O. (1999). p53, p21, Rb, and MDM2 proteins in tongue carcinoma from patients < 35 versus > 75 years. *Oral oncology*, *35*(4), 379–383.
14. Schoelch, M. L., Regezi, J. A., Dekker, N. P., Ng, I. O., McMillan, A., Ziober, B. L., Le, Q. T., Silverman, S., & Fu, K. K. (1999). Cell cycle proteins and the development of oral squamous cell carcinoma. *Oral oncology*, *35*(3), 333–342.
15. Saito, T., Nakajima, T., & Mogi, K. (1999). Immunohistochemical analysis of cell cycle-associated proteins p16, pRb, p53, p27 and Ki-67 in oral cancer and precancer with special reference to verrucous carcinomas. *Journal of oral pathology & medicine : official publication of the International Association of Oral Pathologists and the American Academy of Oral Pathology*, *28*(5), 226–232.
16. Sartor, M., Steingrimsdottir, H., Elamin, F., Gäken, J., Warnakulasuriya, S., Partridge, M., Thakker, N., Johnson, N. W., & Tavassoli, M. (1999). Role of p16/MTS1, cyclin D1 and RB in primary oral cancer and oral cancer cell lines. *British journal of cancer*, *80*(1-2), 79–86.
17. Girod, S. C., Pfeiffer, P., Ries, J., & Pape, H. D. (1998). Proliferative activity and loss of function of tumour suppressor genes as 'biomarkers' in diagnosis and prognosis of benign and preneoplastic oral lesions and oral squamous cell carcinoma. *The British journal of oral & maxillofacial surgery*, *36*(4), 252–260.
18. Gimenez-Conti, I. B., Collet, A. M., Lanfranchi, H., Itoiz, M. E., Luna, M., Xu, H. J., Hu, S. X., Benedict, W. F., & Conti, C. J. (1996). p53, Rb, and cyclin D1 expression in human oral verrucous carcinomas. *Cancer*, *78*(1), 17–23.

Lack of essential data (n=15)

1. Komatsu, M., Saito, K., Miyamoto, I., Koike, K., Iyoda, M., Nakashima, D., Kasamatsu, A., Shiiba, M., Tanzawa, H., & Uzawa, K. (2022). Aberrant GIMAP2 expression affects oral squamous cell carcinoma progression by promoting cell cycle and inhibiting apoptosis. *Oncology letters*, *23*(2), 49.
2. Pennacchiotti, G., Valdés-Gutiérrez, F., González-Arriagada, W. A., Montes, H. F., Parra, J., Guida, V. A., Gómez, S. E., Guerrero-Gimenez, M. E., Fernandez-Muñoz, J. M., Zoppino, F., Carón, R. W., Ezquer, M. E., Fernández-Ramires, R., & Bruna, F. A. (2021). SPINK7 expression changes accompanied by HER2, P53 and RB1 can be relevant in predicting oral squamous cell carcinoma at a molecular level. *Scientific reports*, *11*(1), 6939.
3. Mulder, F. J., Klufah, F., Janssen, F., Farshadpour, F., Willems, S. M., de Bree, R., Zur Hausen, A., van den Hout, M., Kremer, B., & Speel, E. M. (2021). Presence of Human Papillomavirus and Epstein-Barr Virus, but Absence of Merkel Cell Polyomavirus, in Head and Neck Cancer of Non-Smokers and Non-Drinkers. *Frontiers in oncology*, *10*, 560434.
4. Lu, X., Liu, K., Soares, R. C., Thomson, T., Prisman, E., Wu, J., & Poh, C. F. (2018). Potential clinical implications of HPV status and expressions of p53 and cyclin D1 among oropharyngeal cancer patients. *Journal of oral pathology & medicine*, *47*(10), 945–953.

5. Emmett, S., Jenkins, G., Boros, S., Whiteman, D. C., Panizza, B., & Antonsson, A. (2017). Low prevalence of human papillomavirus in oral cavity squamous cell carcinoma in Queensland, Australia. *ANZ journal of surgery*, *87*(9), 714–719.
6. Sannigrahi, M. K., Singh, V., Sharma, R., Panda, N. K., Radotra, B. D., & Khullar, M. (2016). Detection of active human papilloma virus-16 in head and neck cancers of Asian North Indian patients. *Oral diseases*, *22*(1), 62–68.
7. Reyes, M., Rojas-Alcayaga, G., Pennacchiotti, G., Carrillo, D., Muñoz, J. P., Peña, N., Montes, R., Lobos, N., & Aguayo, F. (2015). Human papillomavirus infection in oral squamous cell carcinomas from Chilean patients. *Experimental and molecular pathology*, *99*(1), 95–99.
8. Antonsson, A., Neale, R. E., Boros, S., Lampe, G., Coman, W. B., Pryor, D. I., Porceddu, S. V., & Whiteman, D. C. (2015). Human papillomavirus status and p16(INK4A) expression in patients with mucosal squamous cell carcinoma of the head and neck in Queensland, Australia. *Cancer epidemiology*, *39*(2), 174–181.
9. de Oliveira, M. G., Ramalho, L. M., Gaião, L., Pozza, D. H., & de Mello, R. A. (2012). Retinoblastoma and p53 protein expression in pre-malignant oral lesions and oral squamous cell carcinoma. *Molecular medicine reports*, *6*(1), 163–166.
10. Elango, K. J., Suresh, A., Erode, E. M., Subhadradevi, L., Ravindran, H. K., Iyer, S. K., Iyer, S. K., & Kuriakose, M. A. (2011). Role of human papilloma virus in oral tongue squamous cell carcinoma. *Asian Pacific journal of cancer prevention : APJCP*, *12*(4), 889–896.
11. Weinberger, P. M., Merkley, M., Lee, J. R., Adam, B. L., Gourin, C. G., Podolsky, R. H., Haffty, B. G., Papadavid, E., Sasaki, C., Psyrri, A., & Dynan, W. S. (2009). Use of combination proteomic analysis to demonstrate molecular similarity of head and neck squamous cell carcinoma arising from different subsites. *Archives of otolaryngology--head & neck surgery*, *135*(7), 694–703.
12. Suzuki, H., Sugimura, H., & Hashimoto, K. (2006). p16INK4A in oral squamous cell carcinomas--a correlation with biological behaviors: immunohistochemical and FISH analysis. *Journal of oral and maxillofacial surgery*, *64*(11), 1617–1623.
13. Jayasurya, R., Francis, G., Kannan, S., Lekshminarayanan, K., Nalinakumari, K. R., Abraham, T., Abraham, E. K., & Nair, M. K. (2004). p53, p16 and cyclin D1: molecular determinants of radiotherapy treatment response in oral carcinoma. *International journal of cancer*, *109*(5), 710–716.
14. El-Naggar, A. K., Lai, S., Clayman, G. L., Zhou, J. H., Tucker, S. A., Myers, J., Luna, M. A., & Benedict, W. F. (1999). Expression of p16, Rb, and cyclin D1 gene products in oral and laryngeal squamous carcinoma: biological and clinical implications. *Human pathology*, *30*(9), 1013–1018.
15. Ogawara, K., Miyakawa, A., Shiba, M., Uzawa, K., Watanabe, T., Wang, X. L., Sato, T., Kubosawa, H., Kondo, Y., & Tanzawa, H. (1998). Allelic loss of chromosome 13q14.3 in human oral cancer: correlation with lymph node metastasis. *International journal of cancer*, *79*(4), 312–317.

Overlapping population (n=3)

1. Trivedi, T. I., Tankshali, R. A., Goswami, J. V., Shukla, S. N., Shah, P. M., & Shah, N. G. (2011). Identification of site-specific prognostic biomarkers in patients with oral squamous cell carcinoma. *Neoplasma*, *58*(3), 217–226.
2. Shah, N. G., Trivedi, T. I., Tankshali, R. A., Goswami, J. A., Shah, J. S., Jetly, D. H., Kobawala, T. P., Patel, K. C., Shukla, S. N., Shah, P. M., & Verma, R. J. (2007). Molecular alterations in oral carcinogenesis: significant risk predictors in malignant transformation and tumor progression. *The International journal of biological markers*, *22*(2), 132–143.

3. Pande, P., Soni, S., Kaur, J., Agarwal, S., Mathur, M., Shukla, N. K., & Ralhan, R. (2002). Prognostic factors in betel and tobacco related oral cancer. *Oral oncology*, *38*(5), 491–499.

No oral cancer (n=1)

1. Maiti, G. P., Ghosh, A., Chatterjee, R., Roy, A., Sharp, T. V., Roychoudhury, S., & Panda, C. K. (2012). Reduced expression of LIMD1 in ulcerative oral epithelium associated with tobacco and areca nut. *Asian Pacific journal of cancer prevention : APJCP*, *13*(9), 4341–4346.

Review (n=1)

1. Husain, N., & Neyaz, A. (2017). Human papillomavirus associated head and neck squamous cell carcinoma: Controversies and new concepts. *Journal of oral biology and craniofacial research*, *7*(3), 198–205.

In vitro (n=1)

1. Maiti, G. P., Ghosh, A., Chatterjee, R., Roy, A., Sharp, T. V., Roychoudhury, S., & Panda, C. K. (2012). Reduced expression of LIMD1 in ulcerative oral epithelium associated with tobacco and areca nut. *Asian Pacific journal of cancer prevention : APJCP*, *13*(9), 4341–4346.
